# The fate of electron–hole pairs in polymer:fullerene blends for organic photovoltaics

**DOI:** 10.1038/ncomms12556

**Published:** 2016-09-02

**Authors:** Martina Causa', Jelissa De Jonghe-Risse, Mariateresa Scarongella, Jan C. Brauer, Ester Buchaca-Domingo, Jacques-E. Moser, Natalie Stingelin, Natalie Banerji

**Affiliations:** 1 Department of Chemistry, University of Fribourg, Chemin du Musée 9, CH-1700 Fribourg, Switzerland; 2 Photochemical Dynamics Group, Institute of Chemical Sciences & Engineering and Lausanne Centre for Ultrafast Science (LACUS), Ecole Polytechnique Fédérale de Lausanne (EPFL), SB ISIC GR-MO, Station 6, CH-1015 Lausanne, Switzerland; 3 Department of Materials and Centre for Plastic Electronics, Imperial College London, Exhibition Road, London SW7 2AZ, UK; 4 Division of Physical Sciences and Engineering, King Abdullah University of Science and Technology (KAUST), Thuwal 23955-6900, Saudi Arabia

## Abstract

There has been long-standing debate on how free charges are generated in donor:acceptor blends that are used in organic solar cells, and which are generally comprised of a complex phase morphology, where intermixed and neat phases of the donor and acceptor material co-exist. Here we resolve this question, basing our conclusions on Stark effect spectroscopy data obtained in the absence and presence of externally applied electric fields. Reconciling opposing views found in literature, we unambiguously demonstrate that the fate of photogenerated electron–hole pairs—whether they will dissociate to free charges or geminately recombine—is determined at ultrafast times, despite the fact that their actual spatial separation can be much slower. Our insights are important to further develop rational approaches towards material design and processing of organic solar cells, assisting to realize their purported promise as lead-free, third-generation energy technology that can reach efficiencies over 10%.

## Abstract (web summary)

Charge generation and transport are crucial to the performance of organic solar cells, but the mechanism remains controversial. Causa' *et al*. show that the phase morphology of polymer:fullerene blends determines the exciton dissociation at femtoseconds, although the spatial separation can occur at picoseconds.

## Graphical abstract

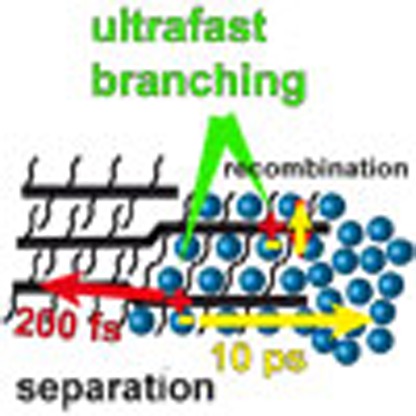

Organic solar cells, often based on thin-film blends of conjugated polymers with fullerene electron acceptors, have reached power conversion efficiencies over 10% (refs 1, 2, 3, 4). Their advantages include low-cost manufacturing, flexibility, light-weight and the possibility for chemical, morphological and photophysical tailoring. However, this tunability makes it difficult to identify all ingredients for high performance—and in particular to simultaneously optimize exciton dissociation to electron–hole pairs, separation of the electron–hole pairs to free charges and transport of the free charges to the electrodes. Lack of physical understanding makes rational control of those processes even more challenging. Especially, the generation mechanism of free charges, which is strongly entangled with short-range charge transport and local structure, has divided the scientific community between views of instantaneous free-charge generation^5^,^6^,^7^,^8^,^9^,^10^,^11^,^12^,^13^, and of slowly separating charge-transfer states^14^,^15^,^16^,^17^. Conflicting conclusions have thereby been drawn from contactless thin-film measurements^6^,^8^,^13^ and studies on full devices under external bias^14^,^16^.

To circumvent these issues, we use here complementary ultrafast pump–probe spectroscopies, based on the evolution of electro-absorption (EA; Stark effect) signatures in the absence and presence of external electric fields. Since the coexistence of intermixed polymer:fullerene regions with neat polymer and fullerene domains is key for high solar cell efficiency^18^,^19^,^20^,^21^,^22^, we place our investigation in the context of different phase morphologies, and selectively target the components of the investigated blends at appropriate excitation wavelengths. The pBTTT:PCBM (poly(2,5-bis(3-hexadecyl-thiophen-2-yl)thieno[3,2-*b*]thiophene:[6,6]-phenyl-C_61_-butyric-acid-methyl-ester) blend is chosen as model system, as this blend gives rise to a well-defined intimately mixed co-crystal phase, whose coexistence with neat domains can be well controlled and structurally characterized^20^,^22^,^23^,^24^.

We directly visualize the motion of excitons and charges in pBTTT:PCBM between the different phases, thus clearly establishing their interplay and their determining role in defining the photophysics of free-charge generation. Importantly, we find that the ability of photogenerated electron–hole pairs to dissociate is already determined at ultrafast times, although the actual spatial separation of the charge pairs can be slower, thus reconciling the two opposing views reported in literature.

## Results

### Transient absorption spectroscopy

**Figure 1:**
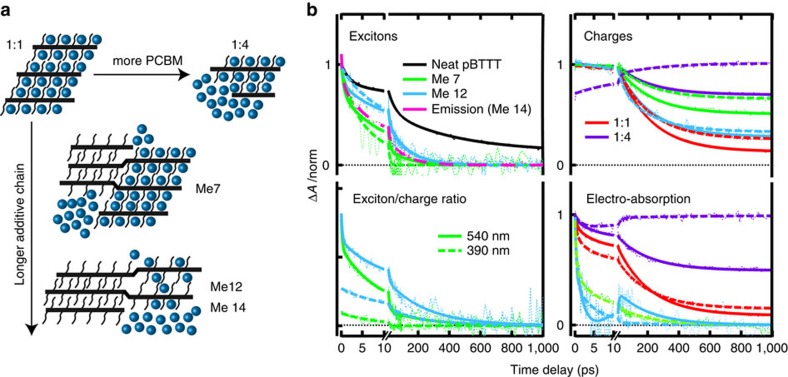
Investigated phase morphologies and TA results. (**a**) Schematic representation of the four investigated phase morphologies of the pBTTT:PCBM blends: fully intercalated (1:1 weight ratio); fully intercalated with neat PCBM clusters (1:4 weight ratio); partially intercalated (1:1 weight ratio processed with Me 7); and predominantly phase-separated (1:1 weight ratio processed with Me 12 or Me 14). (**b**) Normalized dynamics of the isolated populations of the pBTTT excitons, of the charges, of the ratio of the exciton-to-charge population (the absolute value is arbitrary) and of the EA signature, as obtained from the analysis of the TA data. Best sum of exponential fits are superposed to the experimental data. Smooth lines correspond to excitation at 540 nm of the samples indicated in the legend, while dashed lines are for excitation at 390 nm. For the exciton population, the time profile obtained by fluorescence up-conversion spectroscopy for the 1:1 blend processed with Me 14 (predominantly phase-separated) is also shown (500 nm excitation and 800 nm emission).

The phase morphology of the investigated pBTTT:PCBM blends (1:1 weight ratio) is manipulated using fatty acid methyl ester additives of different alkyl chain lengths, from fully intermixed (processed without additives), to partially intercalated (processed with heptanoic acid methyl ester, Me 7), to predominantly phase-separated (processed with dodecanoic or tetradecanoic acid methyl ester, Me 12 or Me 14), as schematically shown in Fig. 1a (refs 23, 24). With excess PCBM (1:4 weight ratio), a further scenario of intermixed co-crystal regions and neat fullerene clusters is achieved^20^,^22^. We present here a new analysis of the femtosecond-resolved transient absorption (TA) spectra^25^, separating their three main contributions: the signatures of pBTTT excitons; of charges (whereby electron–hole pairs and free charges cannot be distinguished); and of the EA (Supplementary Note 1 and Supplementary Figs 1–5). The evolution of the three components is shown in Fig. 1b, and thermal artefacts are discussed in Supplementary Note 2 (with Supplementary Figs 6–8).

### Temporal evolution of the exciton and charge populations

In the contactless TA experiments, pBTTT excitons generated in the co-crystal phase or directly at the interface of neat domains are quenched promptly by electron transfer to PCBM within the ∼100 fs experimental time resolution, since they do not require diffusion before their dissociation^18^,^25^. Therefore, when exciting pBTTT at 540 nm, the signature of pBTTT excitons is only observable in the phase-separated 1:1 blends processed with Me 7 or Me 12, which contain neat pBTTT domains, but not in the 1:1 and 1:4 blends without neat polymer regions (Fig. 1b). The dynamics of the pBTTT excitons in the additive-treated blends decays faster than for neat pBTTT films, due to delayed exciton quenching after diffusion to a PCBM quenching site (time constants of 0.6, 9 and 125 ps). From the average exciton lifetimes, we estimate delayed quenching efficiencies of 88% and 76% in the samples processed with Me 7 and Me 12, respectively (Supplementary Note 3 and Supplementary Figs 9 and 10, prompt quenching is not accounted for). Thus, 12 and 24% of pBTTT excitons generated within the neat polymer domains of the partially phase-separated (Me 7) and predominately phase-separated (Me 12) blends are lost to the ground state. We obtain similar exciton dynamics from time-resolved emission on predominantly phase-separated films (Me 14, Fig. 1b).

To further evaluate how the extent of phase separation affects delayed charge generation, the exciton-to-charge ratio is calculated from the non-normalized pBTTT exciton and charge dynamics. With 540 nm excitation, the initial ratio is the same for the blends processed with Me 7 and Me 12 (Fig. 1b), but it decays more rapidly for the partially phase-separated sample (Me 7), in agreement with higher quenching efficiency and possibly smaller pBTTT domains. When exciting both blends at 390 nm, the dynamics of the delayed exciton quenching is comparable to the one at 540 nm, however, only a very weak signature of pBTTT excitons is present. This is because PCBM is predominantly excited rather than the polymer, and we have previously observed that selective excitation into co-crystal regions at 390 nm can occur as well^25^. Indeed, the initial exciton-to-charge ratio is significantly lower with 390 nm excitation, confirming enhanced prompt charge generation in the intermixed phase. The effect is more pronounced for the partially intercalated Me 7 sample, containing a higher fraction of co-crystal regions.

The evolution of the photo-generated charges in the pBTTT:PCBM blends depends strongly on the phase morphology (Fig. 1b). In most cases, the charge dynamics is dominated by a partial decay with a time constant of 210 ps, which we attribute to geminate charge recombination (gCR), mainly of promptly generated electron–hole pairs in the intermixed regions. When exciting pBTTT at 540 nm, the amplitude of the recombination decreases from 87 (1:1 blend), to 72 (with Me 12), to 49 (with Me 7) and to 30% (1:4 blend). This trend correlates with reported polymer-fullerene contact area (extent of intermixing)^23^, and correlates inversely with solar cell efficiency of corresponding devices^24^. Therefore, not only the presence of neat PCBM clusters (1:4 blend) but also the additive-assisted phase separation in the 1:1 blends (especially with Me 7) prevents gCR and promotes the generation of free, extractable charges. With 390 nm excitation (significant PCBM absorption), the amplitude of gCR in the 1:1 blend (with and without additives) is slightly reduced, possibly because generation of free charges by hole transfer rather than by electron transfer is weakly favoured^26^. In the phase-separated blends (Me 7 and Me 12), the apparent reduction of gCR is caused by a counterbalancing slow rise of the charge signature due to delayed hole transfer from PCBM excitons generated in neat fullerene regions. In the 1:4 sample, the dynamics of the charge population is dominated by a multiphasic rise (sub-picoseconds to hundreds of picoseconds) resulting from this delayed hole transfer from PCBM clusters^27^.

### Evolution of the local EA in the TA spectra

The presence of photo-generated charges in polymer:fullerene films can cause a Stark shift in the absorption of chromophores found in the vicinity of the charges, leading to an EA signature in the TA spectra^6^. The effect probes the local environment of the charges, since it depends on the local electric dipoles around electron–hole pairs and local radial electric fields around free charges. We have previously shown for pBTTT:PCBM that the observed EA signal is associated with transitions in pBTTT and specifically related to the presence of holes in the intermixed co-crystal regions^25^. The EA due to holes in neat pBTTT domains or due to electrons on PCBM has a negligible contribution to the TA spectra.

**Figure 2:**
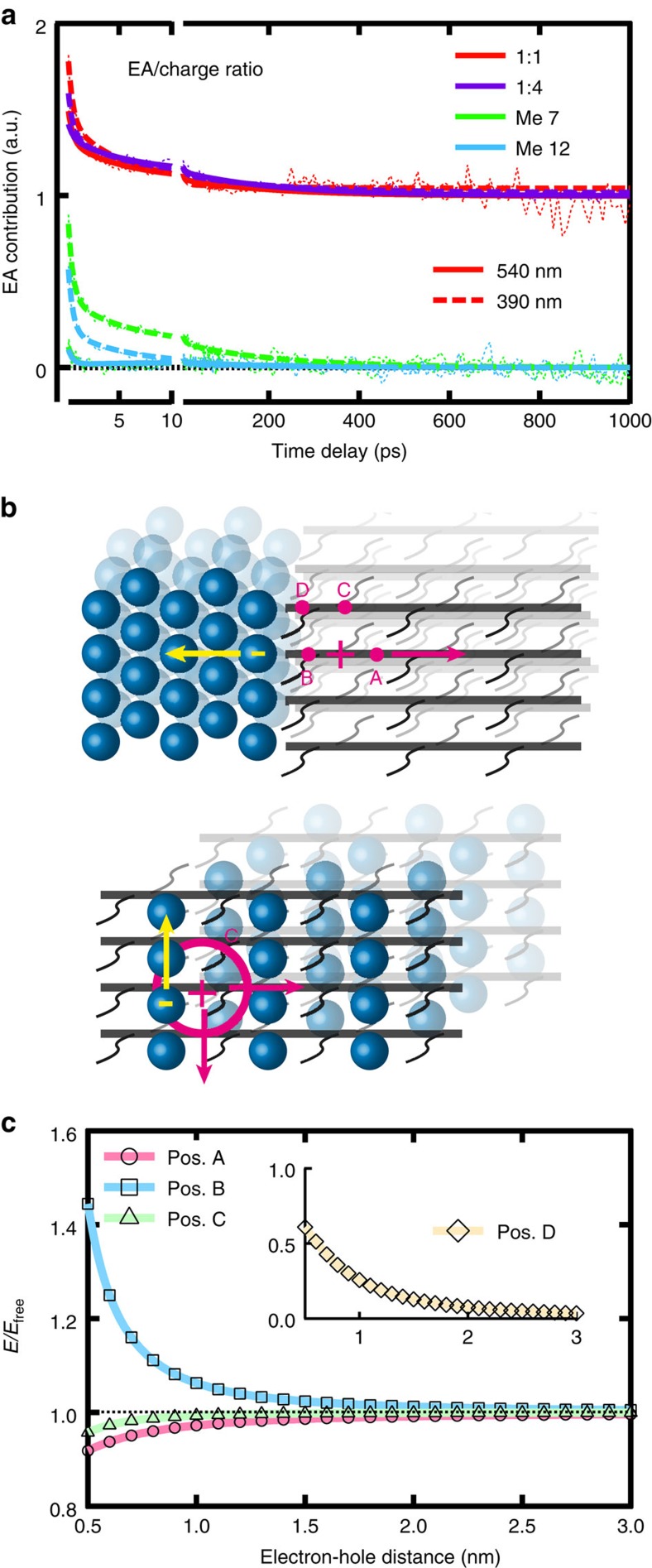
Analysis of the EA signature in the TA data. (**a**) Relative contribution of the EA signature to the charge population, obtained by the analysis of the TA data for the samples shown in the legend (the absolute value is arbitrary). Best sum of exponential fits are superposed to the experimental data. Smooth lines correspond to excitation at 540 nm, while dashed lines are for excitation at 390 nm. (**b**) Schematic three-dimensional representation of the interface between neat pBTTT and neat PCBM domains (top) and of the intercalated pBTTT:PCBM co-crystal region (bottom). Yellow and pink arrows correspond to the motion of the electron and hole, respectively. Different positions of adjacent polymer segments with respect to the electron-hole dipole are indicated (A, aligned with the dipole on the other side of the hole; B, aligned with the dipole between the charges; C, perpendicular to the dipole next to the hole; D, in the central plane dividing the dipole). (**c**) Evolution of the electric field compared with the one around a free charge (*E*/*E*_free_) as a function of the electron–hole distance, felt by polymer segments 0.2 nm away from the hole, located at the different positions indicated in **b**, as obtained by an electrostatic point charge model.

In the 1:1 and 1:4 blends processed without additives (where all pBTTT is present in co-crystal regions), the evolution of the EA signature is strongly correlated to the dynamics of the charge population (Fig. 1b). For example, it decays together with gCR with 540 nm excitation, and slowly increases with delayed hole transfer in the 1:4 blend with 390 nm excitation. To have a better appreciation of the EA contribution without the effect of the charge dynamics, the EA-to-charge ratio is calculated (Fig. 2a). For the 1:1 and 1:4 blends, the evolution of this ratio is identical, and it is also very similar for 540 and 390 nm excitation. About one-third of the relative EA contribution decays with time constants of ∼0.5 and 5.5 ps. This fast decay is likely caused by relaxation and localization of the holes on the polymer chains (cooling and geometric self-trapping), since a higher initial delocalization plausibly increases the locally induced Stark effect at short times. In agreement, the initial EA contribution is slightly higher with excess energy excitation at 390 nm. Initial exciton delocalization has been reported in conjugated polymers^28^. Possibly, prompt exciton dissociation in the intermixed phase of pBTTT:PCBM is faster than exciton localization, generating initially highly non-relaxed charges.

In the absence of neat pBTTT domains in the 1:1 and 1:4 blends, the photo-generated holes necessarily remain in the co-crystal regions until they recombine, causing a strong local Stark effect even at long times. Therefore, apart from the fast relaxation, the EA-to-charge ratio remains constant within the 1 ns time window of the experiment (Fig. 2a). The expected electron migration to the neat PCBM clusters, present only in the 1:4 blend, does not affect the evolution of the EA compared with the fully intercalated 1:1 sample. This is in contrast to other reported systems^6^, but can be reconciled by considering the very different electrostatic situations at the interface of phase-pure domains and at the molecular interface in the intermixed co-crystal phase (Fig. 2b)^29^. The closest neighbouring polymer segments, which dominate the EA response in the co-crystal regions, are located on both sides of the hole on the same chain, and on the chains *π*-stacked above and below the hole, thus perpendicular to the electron–hole dipole (position C, bottom of Fig. 2b). An electrostatic simulation of the electric field in this position confirms that its magnitude is quite insensitive to electron–hole distance, and essentially equal to the field generated around a free charge for an electron–hole separation >0.8 nm (Fig. 2c, Supplementary Note 4 and Supplementary Fig. 11). In contrast, polymer segments in positions A–D are affected by the electric field of a dipole separating across a domain interface (top of Fig. 2b), causing stronger dependence of the EA on electron–hole distance.

With phase separation in the 1:1 blends processed with Me 7 and Me 12, we find that over 85% of the absolute EA signature vanishes within a few picoseconds (mainly with a <1 ps time constant), for both 540 and 390 nm excitation (Fig. 1b). The charges present at long time delays cause no significant EA contribution to the TA spectra, because the holes have migrated to neat pBTTT domains, where they give rise to a negligible Stark effect. We therefore attribute the fast decay of the EA amplitude to transport of promptly generated holes from co-crystal regions to neat pBTTT domains in the phase-separated blends. The observed weak decay of the remaining EA with 210 ps is due to residual charges in the intermixed regions that undergo gCR. For 540 nm excitation, we assign the observed ∼10 ps rise of the EA (shown here for Me 12) to excitons from neat domains diffusing to co-crystal regions and dissociating there. Scaling the dynamics of the EA signature by the charge population (Fig. 2a) reveals a strongly enhanced EA contribution for 390 nm excitation, especially for the partially phase-separated blend processed with Me 7. This confirms selective excitation of co-crystal regions at 390 nm. Even then, the initial EA contribution is smaller than for the 1:1 and 1:4 blends without neat pBTTT domains, since prompt charge generation in the phase-separated blends can also occur at the interfaces of neat domains, causing negligible EA.

### Electromodulated differential absorption spectroscopy

**Figure 3:**
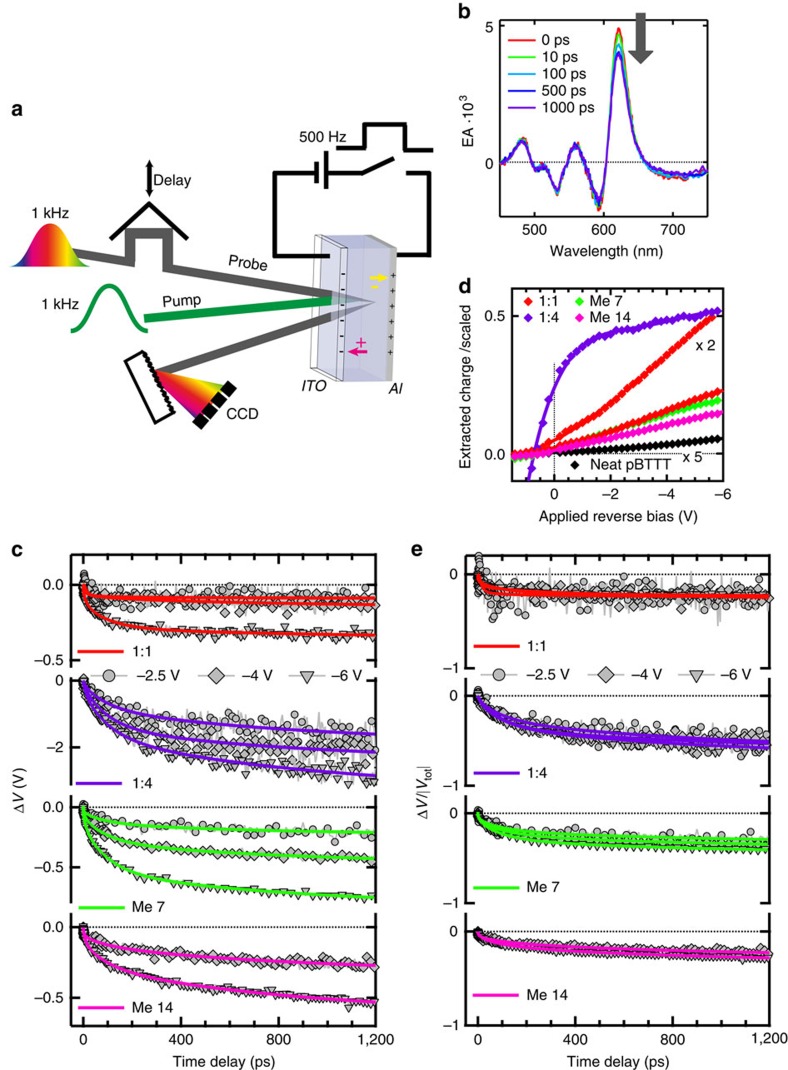
Electromodulated differential absorption set-up and results. (**a**) Schematic representation of the set-up used for time-resolved EDA spectroscopy on full solar cell devices under reverse bias. (**b**) Typical decay of the EA spectrum obtained for pBTTT:PCBM (1:1 by weight, processed with Me 7, −6 V applied bias) at different time delays after excitation at 540 nm. (**c**) Voltage drop dynamics (Δ*V*) obtained from the EDA decay around 620 nm for the investigated pBTTT:PCBM devices, at reverse biases of −2.5 (circles), −4 (diamonds) or −6 V (triangles), with excitation at 540 nm at a fluence of ∼1.0 μJ cm^−2^. Solid lines are best sum of exponential fits. (**d**) Extracted photo-generated charge (scaled by the number of absorbed photons) as a function of applied reverse bias, obtained by integrating the photocurrent transients measured with an oscilloscope via a 50 Ω series load, after exciting the different solar cells (see legend) at 540 nm at a fluence within the linear photocurrent regime. The pBTTT:PCBM (1:1 by weight) data are shown twice, once scaled for better comparison with the pBTTT:PCBM (1:4 by weight) data. (**e**) Voltage drop dynamics for the same devices as in **c**, normalized by the total voltage drop calculated from the device capacitance and extracted photo-generated charge (Δ*V*/|*V*_tot_|).

To learn more about the transport of charges in the pBTTT:PCBM blends with different phase morphologies, we exploit the Stark effect in a complementary way to the TA measurements. We externally apply a reverse bias to full solar cell devices containing the pBTTT:PCBM blends as active layer, establishing a homogeneous electric field of the order of 10^5^ V cm^−1^. The current response of the devices is recorded in parallel to the spectroscopic measurements (Supplementary Note 5 and Supplementary Figs 12 and 13). The difference in the absorption spectrum in the presence and absence of the field, recorded in reflection mode using a femtosecond probe beam (Fig. 3a), yields the EA spectrum of the bulk organic material (Supplementary Fig. 14), in contrast to the local EA contribution seen in the TA spectra. The bulk EA spectra of the different blends are related to the first and second derivatives of the absorption spectrum (Supplementary Note 6 and Supplementary Fig. 15), as expected for a Stark effect originating from changes in polarizability and dipole moment between the ground and excited states^30^. For time-resolved electromodulated differential absorption (EDA) spectroscopy^14^,^31^,^32^,^33^, we measure the EA spectra after excitation of the devices with femtosecond pump pulses. When the photo-generated charges are transported and extracted at the electrodes, this shields the externally applied reverse bias and thus reduces the electric field within the active layer of the devices, directly resulting in a decrease of the EA amplitude at different time delays after excitation (Fig. 3b). Knowing the typically quadratic relation between the EA amplitude and the applied voltage, the decrease of the EA signal is translated to a femtosecond-resolved voltage drop across the solar cells (Supplementary Note 7 and Supplementary Figs 16 and 17).

Unlike TA spectroscopy, EDA is selective to electron–hole pairs that spatially dissociate to free charges and are subsequently transported to the electrodes. Neutral excitons and those geminate charge pairs, which undergo gCR in 210 ps without dissociating, are invisible. The time-resolved voltage drops obtained following excitation at 540 nm of all investigated pBTTT:PCBM blends are strongly multiphasic (with time constants ranging from a few to hundreds of picoseconds, Fig. 3c), reflecting the complexity of potentially three-dimensional electron and hole transport in the disordered materials^34^. At an applied reverse bias of −6 V and for comparable excitation densities, the voltage drop on the 1 ns timescale is ∼−0.3 V in the 1:1 blend (only fully intercalated phase with important gCR) and increases to ∼−2.8 V in the 1.4 blend (where neat PCBM clusters prevent gCR in the co-crystal regions). This indicates that the magnitude of the voltage drop scales with the ability of a given phase morphology to generate free charges. Since the experiments are carried out under reverse bias and at low pump fluence (linear photocurrent regime, Supplementary Fig. 13), we can assume that all free charges (electrons and holes) are successfully extracted without undergoing bimolecular recombination^35^. The extracted photo-generated charge (from integrated photocurrent transients measured in parallel to the EDA experiments, Fig. 3d) therefore provides a direct measure of the number of free charges in the investigated devices.

To isolate the effect of charge transport in the voltage drop dynamics from the total yield of photo-generated free charges, the EDA data are normalized according to the extracted charge and device capacitance (Fig. 3e, Supplementary Note 7 and Supplementary Fig. 18). The normalized voltage drop is expected to be −1 after full charge extraction, but typically does not reach this value on the 1 ns timescale. From this, it can be estimated that about 20, 30 and 60% of the free charges are extracted within 1 ns in the fully intercalated 1:1 blend, the phase-separated additive samples and the intercalated 1:4 blend with PCBM clusters, respectively (Fig. 3e). This is consistent with previous TREFISH (time-resolved electric-field-induced second harmonic) generation and time-of-flight measurements on polymer:fullerene blends, showing that part of the charges are extracted within 3 ns, while a large fraction (>50%) of trapped charges is extracted on a much slower hundreds of nanosecond timescale^32^. The trapped charges are likely initially free electrons and holes that become trapped during transport, but we cannot exclude a contribution of long-lived geminate charge pairs^36^. We observe for pBTTT:PCBM that the fraction of trapped charges is enhanced in the co-crystal phase (1:1 blend), but is also surprisingly high in the phase-separated blends treated with additives, with respect to the 1:4 sample containing neat fullerene but no pBTTT domains.

**Figure 4:**
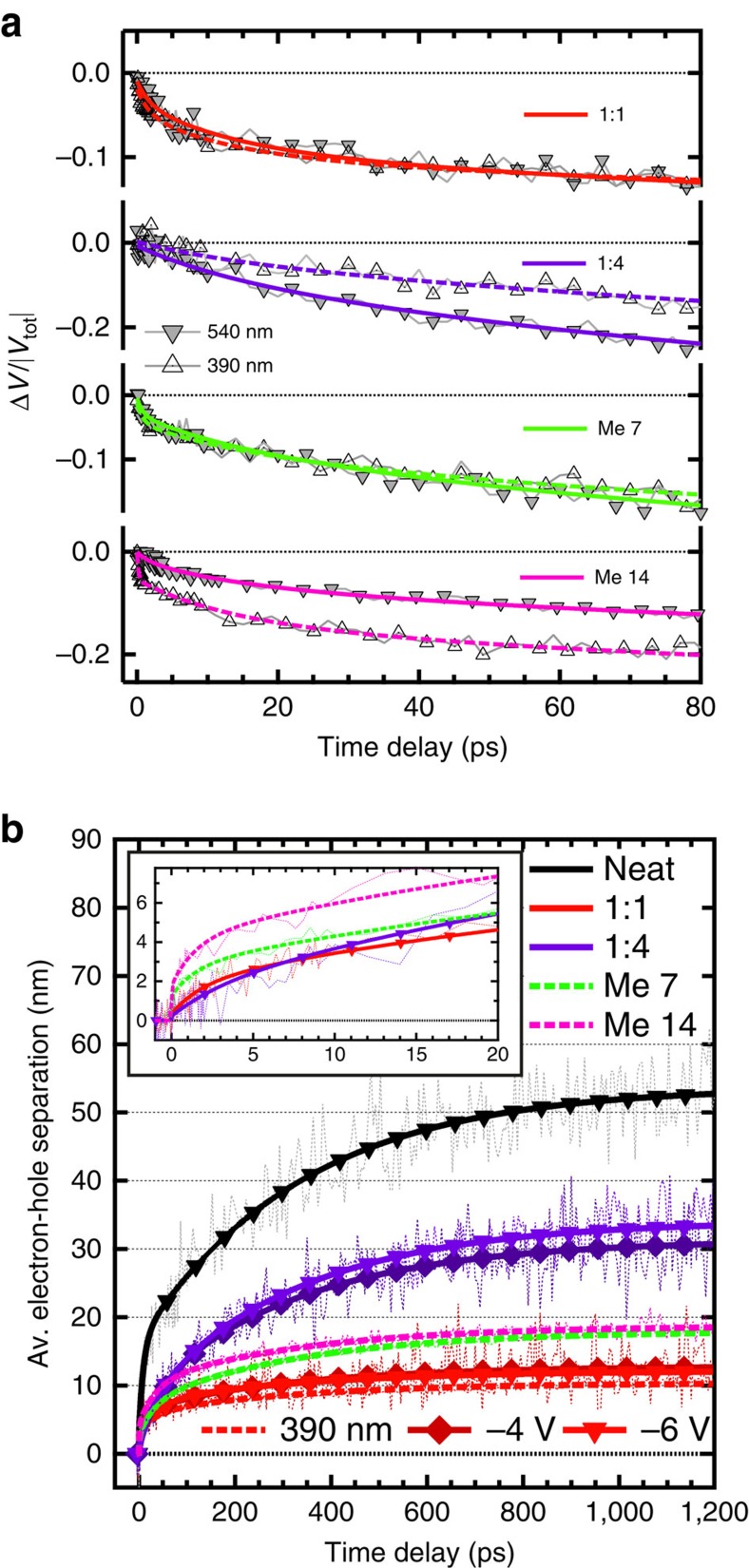
Analysis of the EDA data. (**a**) Normalized voltage drop dynamics (Δ*V*/|*V*_tot_|) obtained from the EDA decay for the different investigated pBTTT:PCBM devices (see legend), at a reverse bias of −6 V, with excitation at 390 (empty triangles, dashed fit curve) or 540 nm (full downward triangles, smooth fit curve) at a fluence within the linear photocurrent regime. (**b**) Average electron–hole separation calculated from the voltage drop dynamics across the devices shown in the legend, at an applied bias of −6 (full downward triangles) or −4 V (full diamonds). Dashed lines are for 390 nm excitation (at −6 V) and smooth lines for 540 nm excitation. The inset zooms on the first 10 ps. Best sum of exponential fits are shown as thick lines.

The normalized voltage drop for excitation wavelengths of 540 and 390 nm is similar in the fully intercalated 1:1 blend and the partially phase-separated blend processed with Me 7 (Fig. 4a). However, the voltage across the cell decreases more slowly with 390 nm than with 540 nm excitation in the 1:4 sample, which is related to delayed hole transfer occurring from excitons generated within PCBM clusters. The transport of the charges starts later, after their delayed generation, leading to the observed delayed voltage drop. In the predominantly phase-separated pBTTT:PCBM blend processed with Me 14, the voltage drop is this time faster with 390 nm excitation, and shows an ultrafast ∼200 fs component. This confirms our interpretation of the TA data, where we assign a similarly fast decay of the local EA signature to selective excitation into co-crystal regions at 390 nm, followed by migration of the holes to neat pBTTT domains on the <1 ps timescale.

### Average electron–hole separation

We obtain more insight about the transport of charges as a function of phase morphology by calculating the average electron–hole separation from the normalized voltage drop dynamics. The analysis yields the sum of the electron and hole motion in the direction towards the electrodes, and is only valid assuming prompt (not delayed) charge generation (Supplementary Note 8). Although the free-charge yield in the neat pBTTT device is extremely low^37^, those charges (predominantly the holes) are transported very efficiently to an average electron–hole separation of ∼50 nm on the 1 ns timescale (Fig. 4b). The transport is slower in all pBTTT:PCBM blends, indicating a negative effect of PCBM intercalation, in agreement with reported local mobilities measured by time-resolved microwave conductivity^38^. Although we cannot infer the initial separation of the charge pairs (not necessarily oriented along the probed direction), it is unlikely higher than 1 nm in the particular geometry of the co-crystal phase. In both the fully intercalated 1:1 blend and the 1:4 sample with additional neat PCBM domains, the dissociating charge pairs then separate by a distance of about 3 nm within 10 ps, which we assign to initial transport within the co-crystal regions (Fig. 4b). After 10 ps, the electron–hole separation is strongly enhanced only in the 1:4 blend and reaches ∼30 nm within 1 ns. We interpret this with electrons reaching the neat PCBM clusters after 10 ps, where their transport is strongly favoured. Improved transport in the 1:4 blend, dominated by electron mobility, has been reported on both the local and macroscopic length scale^39^,^40^. In contrast, a separation of only ∼10 nm is achieved within 1 ns for the 1:1 blend, as the electrons are trapped in the co-crystal regions. For the partially or predominantly phase-separated blends processed with Me 7 or Me 14 and excited selectively into the co-crystal phase at 390 nm, an ultrafast electron–hole separation of ∼2 nm occurs within only 200 fs (inset, Fig. 4b). As discussed before, this is due to ultrafast hole transport from the co-crystalline to neat pBTTT regions.

From the initial slope of the time-dependent electron–hole separation (Fig. 4b)^16^, we estimate a charge mobility on the 2 nm length scale of the order of 10^−1^ cm^2^ V^−1^ s^−1^ for the 1:1 and 1:4 blends (pBTTT present only in the co-crystal phase), and of 1–2 cm^2^ V^−1^ s^−1^ for the phase-separated samples processed with additives. This is notably higher than reported macroscopic mobility (∼10^−3^ to 10^−4^ cm^2^ V^−1^ s^−1^)^40^, and even local microwave conductivity mobility (∼10^−2^ cm^2^ V^−1^ s^−1^) in pBTTT:PCBM blends. The main reason for high mobility on the nanometre length scale is that charges are statistically less likely to encounter grain boundaries or traps^34^,^41^. Moreover, it is probable that the ultrafast hole transport in the phase-separated samples occurs along pBTTT chains connecting the intermixed to neat polymer domains (tie molecules), assisted by high backbone mobility and an energy offset between the two phases^15^,^34^,^42^. From the relaxation of the EA contribution in the TA data, we deduce higher initial (<1 ps) delocalization of the charges, which might also assist the fast hole transport. In spite of this initial ultrafast transport, the charges in the phase-separated samples then slow down and separate only to about ∼20 nm within 1 ns (compared with ∼30 nm in the 1:4 blend), indicating enhanced charge trapping in the neat phases obtained by the use of processing additives (Fig. 4b). In agreement, the mobility of the Me 7 and Me 12 samples measured by time-resolved microwave conductivity is in-between that of the 1:1 and the 1:4 blends^24^,^39^.

### Effect of applied bias on free-charge generation

Finally, measurements on full devices provide us with the opportunity to investigate the bias-dependence of charge generation, recombination and transport. Both the absolute EDA voltage drop (Fig. 3c) and the extracted charge from the integrated photocurrent (Fig. 3d) increase with the applied reverse bias (540 nm excitation). In the relevant −2 V to −6 V range, this increase is more pronounced for the phase morphologies subject to gCR and charge trapping (fully intercalated 1:1 blend and phase-separated additive samples), than in the 1:4 blend, where the neat PCBM clusters assist free-charge generation and transport. A similar voltage dependence for the 1:1 and 1:4 blends has been reported using the time-delayed collection field method^43^. It suggests that applying a reverse bias helps to separate geminate charge pairs that would otherwise undergo gCR, and/or helps to prevent trap-based recombination^44^,^45^. Since the 1:4 sample already shows higher solar cell efficiency, better transport, reduced recombination and intrinsically more efficient free-charge generation, less additional improvement is obtained at high reverse bias. With 390 nm excitation, the trends are similar (Supplementary Fig. 13, the apparent increase of extracted charge is an artefact due to the spectral dependence of cavity interference effects)^46^.

Strikingly, we observe a negligible dependence of the normalized voltage drop on the applied reverse bias for all pBTTT:PCBM blends (Fig. 3e). This implies that although the absolute number of extractable charges increases with reverse bias, there is a weak effect on the transport of the electrons and holes towards the electrodes. This picture is further supported by the time-resolved average electron–hole separation, which shows no significant bias-dependence (Fig. 4b). An explanation is that the field-independent transport is dominated by diffusion, rather than by drift in an electric field, as was already reported for other polymer:fullerene blends^16^. Moreover, the separation distance calculated with our approach does not represent the separation of individual, randomly oriented electron–hole pairs undergoing transport in all spatial directions, but rather the projection of the average motion of the charges onto the axis joining the electrodes. Charge transport in pBTTT is essentially two-dimensional (along the polymer backbones and in the *π*-stacking direction, Fig. 2b)^47^, with higher mobility along the backbones due to paracrystalline disorder in the *π*-stacks^34^,^48^,^49^. The motion of electrons along PCBM percolation paths in the co-crystal regions is also limited to one dimension (Fig. 2b)^29^. Therefore, the preferred pathways for charge transport are not necessarily aligned with the applied external field, contributing to apparent bias-independence of the transport.

In EDA spectroscopy, the dynamics of the voltage drop is also related to the generation rate of extractable charges, if this is not prompt. Therefore, we expect that slow bias-induced dissociation of electron–hole pairs (delayed free-charge generation) is revealed by additional slow components in the time-resolved voltage drop at high applied fields. However, we see no such difference in the dynamics within the measured time window, only the amplitude increases with external bias (Fig. 3c). Together with the fact that the average electron–hole distance is field-independent (Fig. 4b), this suggests that the external reverse bias does not impact the dissociation of charge pairs on the 0.1–1,000 ps timescale. It can be argued that it enhances the extraction of trapped charges within hundreds of nanoseconds^47^. However, this is unlikely the main cause for increased photocurrent yield at high bias, since mainly the extent of fast gCR (210 ps), not of slow trap-based recombination, determines solar cell efficiency in pBTTT:PCBM blends.

Moreover, when considering the voltage drop dynamics without normalization (Fig. 3c), the amplitude of the decay increases with bias even at the earliest measurable times. This implies that the effect of the external field is to generate a greater pool of dissociating charges, leading to an instantaneously enhanced EDA response, within the (<100 fs) time resolution of the experiment. Similarly, the absolute voltage drop within the first picosecond is clearly higher in the 1:4 blend (where PCBM clusters prevent gCR) compared with the 1:1 blend (where gCR in the co-crystal phase is dominant). This shows that increased free-charge generation in the presence of the neat domains is not only related to better transport, since the average electron–hole separation in the two samples only diverges after 10 ps, once the electrons have reached the PCBM clusters (Fig. 4d). We conclude that the branching between charge pairs destined for gCR and those destined to separate is determined at ultrafast times, at the moment of exciton dissociation. Both the application of an external field, and the proximity of neat phase domains shift the branching ratio towards free-charge generation.

## Discussion

The generation mechanism of free, extractable charges in polymer:fullerene blends is still highly debated in the scientific community, with opposing views of, on the one hand, instantaneous free-charge generation^5^,^6^,^7^,^8^,^9^,^10^,^11^,^12^,^13^, and on the other hands, of slowly separating charge transfer states^14^,^15^,^16^,^17^. Our results reconcile those two views. They show that the ability of electron–hole pairs to dissociate is largely determined at ultrafast times, while the actual spatial separation of the charges can occur more slowly and depends on the transport properties in a given phase morphology. The instantaneous fate of photo-generated charge pairs has been previously explained by ultrafast long-range charge separation, delocalization into neat domains and/or involvement of hot states^5^,^6^,^7^,^8^,^9^,^10^,^11^,^12^,^13^,^45^. The latter has subsequently been shown to play a minor role^17^. Moreover, for charge pairs generated within co-crystal regions of pBTTT:PCBM, there is structurally little opportunity for ultrafast long-range charge separation or for delocalization into (not necessarily directly adjacent) neat PCBM or pBTTT regions (Fig. 2b). These mechanisms likely play a more important role to separate charges across neat domain interfaces, rather than in situations involving an intermixed phase.

We therefore propose that relatively subtle changes in the molecular arrangement (donor-acceptor separation and orientation) and local environment (disorder, microscopic fields, delocalization and energy gradients) of the initially generated electron–hole pairs are sufficient to determine whether the geminate charges will be able to separate or not^42^,^50^,^51^,^52^. Those parameters affect mainly the electronic coupling in the electron–hole pairs, which establishes the rate of back-electron transfer (recombination) to the ground state. Thus, we hypothesize that two distinct pools of electron–hole pairs with different electronic couplings are generated directly after exciton dissociation, whereby the more tightly bound ones geminately recombine (as seen by TA spectroscopy), whereas the loosely bound ones dissociate to free charges (leading to the observed EDA decay). This is reminiscent of the situation reported for tight and loose ion pairs generated by bimolecular electron transfer in solution^53^. We find here that the proximity of neat regions to intermixed phases can affect the local environment in the latter (electric fields, energy gradients and delocalization) and thus the nature of the electron–hole pairs at the moment of their generation, influencing their intrinsic ability to dissociate. Similarly, improved ability to dissociate can be achieved by applying an external reverse bias, especially for poorly performing phase morphologies. The existence of charge transfer states with different abilities to dissociate, depending on the local environment, has also been reported in a photo- and electro-luminescence study of various polymer:fullerene blends^54^.

For the dissociating electron–hole pairs, we comprehensively map out the pathways of electrons and holes between the different phases of the pBTTT:PCBM blends. For charges generated within the co-crystal regions, it takes the electrons ∼10 ps to arrive in the neat PCBM clusters, while the holes reach the neat pBTTT domains already in <200 fs (assisted by high intrachain mobility, energy offsets between the two phases and early delocalization). Once in the neat pBTTT or PCBM domains, the charges can travel further on the investigated 1 ns timescale than if they remain in the co-crystal phase, in agreement with better charge transport (and polaron delocalization)^55^ in neat pBTTT domains, as well as with less dispersive transport in neat PCBM regions^32^. Nevertheless, in spite of high initial mobilities and of the beneficial impact of neat phases on the spatial separation of the dissociating electron–hole pairs^15^,^22^,^32^,^56^, we find that the transport is highly dispersive in all the investigated blends (that is, slows down with increasing distance as charges become increasingly trapped)^34^,^41^,^57^. As a consequence, it takes about 10–20 ps in all the investigated blends to reach the ∼5 nm electron–hole separation necessary to overcome their Coulomb interaction, in agreement with previously reported slow dissociation to free charges^15^,^16^,^17^. This an order of magnitude faster than gCR (210 ps), but at least two orders of magnitude slower than the ultrafast <100 fs branching to recombining or dissociated charge pairs.

In conclusion, we answer the long-standing question of how free charges are generated in polymer:fullerene blends, and how the different steps, from exciton dissociation via charge pair separation to transport, depend on phase morphology and applied bias. We demonstrate that the fate of photogenerated charge pairs—whether they will geminately recombine or separate to free charges—is determined at sub-100 fs times, even if the actual spatial separation of the charges can be considerably slower. We focus here on the pBTTT:PCBM model system, but our conclusions are generally relevant, since the interplay of neat and intermixed phases in donor:acceptor blends is a general issue in polymer:fullerene blends and, likely, most organic photovoltaic systems^18^,^19^,^20^,^21^,^22^. In particular our insights concerning the impact of the molecular arrangement, electronic coupling and local environment in determining the ability of electron–hole pairs to dissociate will be important to further develop rational approaches towards materials design and processing with control of the donor:acceptor interface at molecular level.

## Methods

### Sample preparation

The synthesis of pBTTT (Mn=34 kDa, Mw=66 kDa), as used in this study, has been previously reported^58^. We purchased PCBM from Solenne, and the heptanoic acid methyl ester (Me 7), dodecanoic acid methyl ester (Me 12) and tetradecanoic acid methyl ester (Me 14) from Aldrich and Fluka. They were used without further purification. We prepared solutions of neat pBTTT, pBTTT:PCBM (1:1 or 1:4 weight ratio) and pBTTT:additive:PCBM (1:1 polymer:fullerene weight ratio with 10 molar equivalents of the additive per monomer unit of the polymer) in 1,2-ortho-dichlorobenzene. The concentration of pBTTT was 10 mg ml^−1^ in all solutions, which we left stirring for more than 4 h at 100 °C to ensure complete dissolution of the materials. We then deposited films on glass or patterned indium tin oxide (ITO) substrates by wire-bar coating from hot solutions (∼85–90 °C) onto substrates kept at either room temperature (neat pBTTT and additive samples) or 35 °C (1:1 and 1:4 blends). For the solar cell devices, aluminium (Al) counter electrodes were thermally evaporated on top of the polymer:fullerene layer, yielding an active area between the ITO and Al electrodes of 0.1 or 0.2 cm^2^. Oxygen was removed from the films by keeping them for 24 h in vacuum and then sealing them inside the glovebox. The film thickness was 100–110 nm with a maximum absorption in the visible of about 0.5 (PerkinElmer Lambda 950 spectrophotometer).

### Transient absorption and EDA spectroscopy

The set-ups for femtosecond TA and EDA spectroscopy were closely related and based on a pump–probe scheme that we have previously described^25^,^59^. In brief, we used an amplified Ti:sapphire laser system (CPA-2001, Clark-MXR) with a 1 kHz repetition rate and output wavelength of 780 nm. To excite the samples, which consisted of just the polymer:fullerene films on glass substrates for TA spectroscopy and of full devices for EDA spectroscopy, the pump beam was either at 390 nm (frequency-doubled laser output, ∼150 fs pulse duration) or at 540 nm (generated with a commercial two-stage non-collinear optical parametric amplifier (NOPA, Clark-MXR), ∼50–60 fs pulse duration). We determined the pump diameter at the sample to be 0.8 mm for the TA and 3.45 mm for the EDA experiments, with a Thorlabs beam profiler (BC106-Vis, 1/*e*^2^ cutoff). For the EDA measurements, the beam homogeneously covered the entire active area of the device. The fluence (<5 μJ cm^−2^) was adjusted to be in the linear regime for TA intensity or photocurrent, and no sample degradation was observed^25^.

To probe the samples in TA and EDA spectroscopy, we generated a white light beam by passing part of the 780 nm laser output through a 5 mm thick moving CaF_2_ plate (for TA, 400–1,050 nm), or through a 3 mm sapphire disc (for EDA, 400–750 nm). The remaining fundamental was removed with appropriate filters. The pump and probe beams (at magic angle relative polarization) were spatially overlapped in the sample and time delayed with respect to one another using a computer-controlled translation stage. After being transmitted through the TA sample, or being reflected off the aluminium electrode of the EDA device (which was entered through the transparent ITO side), the probe beam was dispersed in a grating spectrograph (SpectraPro 2500i, Princeton Instruments or SR163, Andor Technology) and finally detected shot-by-shot at 1 kHz rate with a 512 × 58 pixel back-thinned charge-coupled device (Hamamatsu S07030-0906, assembled by Entwicklungsbüro Stresing). To correct shot-to-shot fluctuations, we split part of the probe beam before the sample to a reference beam, reaching a second detector.

For TA spectroscopy, the pump pulses were modulated (chopped at half the amplifier frequency, 500 Hz), so that the transient spectra could be computed from the difference of the transmitted probe intensity when the pump was on and off. On the other hand, for EDA spectroscopy, the pump was always on (no chopper) and we modulated instead the electric field applied across the contacts of the solar cell at half the amplifier frequency (500 Hz), using square voltage pulses provided by a synchronized function generator (Tektronix AFG 2021, up to −6 V reverse bias, 100 μs pulse duration). We thus calculated the EDA spectra as the absorption difference between the excited sample with and without applied bias. To obtain steady-state EA) spectra, the pump beam was blocked. For both TA and EDA, the differential spectra were averaged over 3,000–5,000 shots and recorded as a function of pump–probe delay (up to 1.2 ns). In parallel to the optical EDA measurements, the current response across the used devices was recorded via a 50 Ω series load with a 400 MHz oscilloscope (Tektronix TDS 3044B).

### Fluorescence up-conversion spectroscopy

The set-up for fluorescence up-conversion measurements was based on a modified FOG100 system (CDP Lasers & Scanning Systems). It used the 1,000 nm output of a Ti:sapphire oscillator (Mai Tai HP, Spectra-Physics, 80 MHz repetition rate, 100 fs pulse duration), which was frequency-doubled for sample excitation at 500 nm. Encapsulated thin-film samples (not full devices) were measured and constantly rotated to avoid degradation. We used a pump intensity per pulse of about 4 mW, yielding a fluence of 3 μJ cm^−2^, with a spot diameter of 50 μm (any intensity effects were negligible). To detect the sample fluorescence, it was up-converted by sum-frequency generation with a time-delayed gate beam in a BBO crystal, before being dispersed in a monochromator and detected with a photomultiplier tube operating in the photon-counting mode. We tuned the phase-matching conditions when recording the fluorescence dynamics at different emission wavelengths and used magic angle polarization between the pump and gate pulses.

### Data availability

The authors declare that all data supporting the findings of this study are available from the corresponding author on request.

## Additional information

**How to cite this article:** Causa, M. *et al*. The fate of electron–hole pairs in polymer:fullerene blends for organic photovoltaics. *Nat. Commun.* 7:12556 doi: 10.1038/ncomms12556 (2016).

## Supplementary Material

Supplementary Information Supplementary Figures 1-18, Supplementary Notes 1-8 and Supplementary References
