# Supplementary material for: The fate of electron–hole pairs in polymer:fullerene blends for organic photovoltaics
Source: Nat Commun. 2016 Sep 2;7:12556. doi: 10.1038/ncomms12556 (PMC5025766; doi:10.1038/ncomms12556)
Supplement: Supplementary Information — Supplementary Figures 1-18, Supplementary Notes 1-8 and Supplementary References [file ncomms12556-s1.pdf]

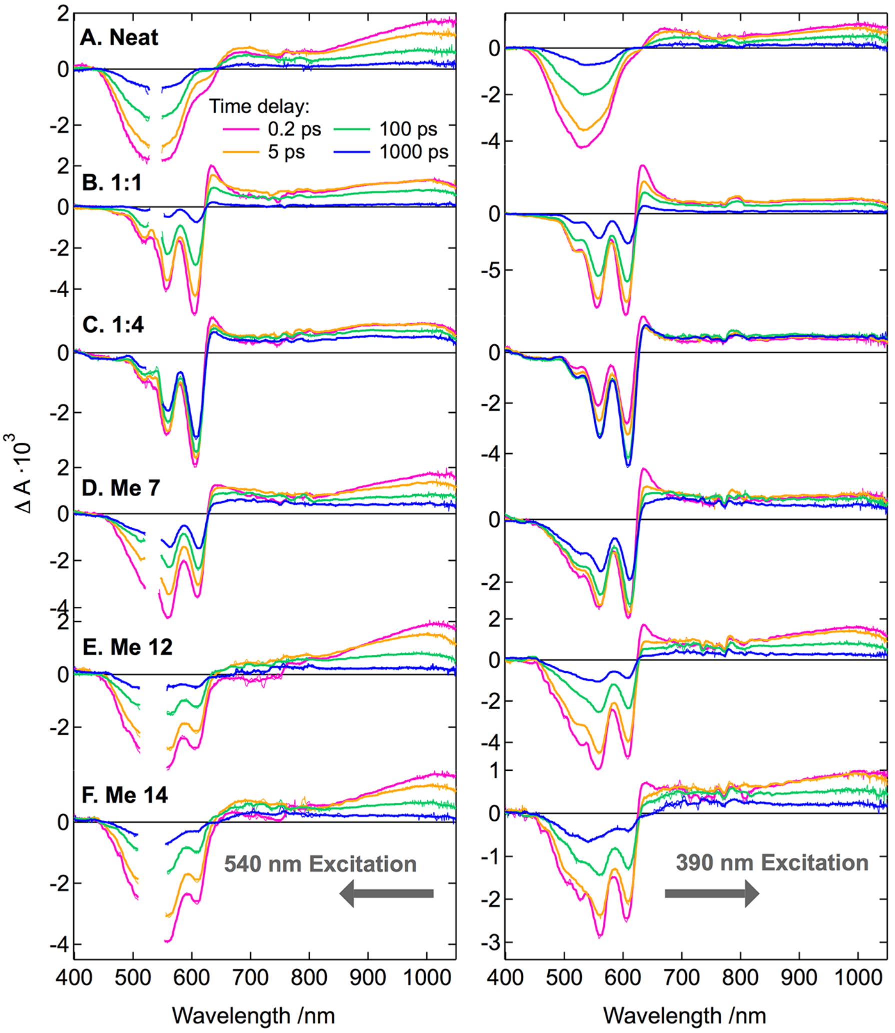

Supplementary Figure 1. Transient absorption spectra at selected time delays after excitation. A) Neat pBTTT, B) pBTTT:PCBM (1:1 weight ratio), C) pBTTT:PCBM (1:4 weight ratio), D) pBTTT:PCBM (1:1, processed with Me 7), E) pBTTT:PCBM (1:1, processed with Me 12) and F) pBTTT:PCBM (1:1, processed with Me 14). The spectra on the left side were recorded following excitation at 540 nm, and the ones on the right side following excitation at 390 nm. Thicker solid lines were smoothed and overlaid to the raw experimental data.

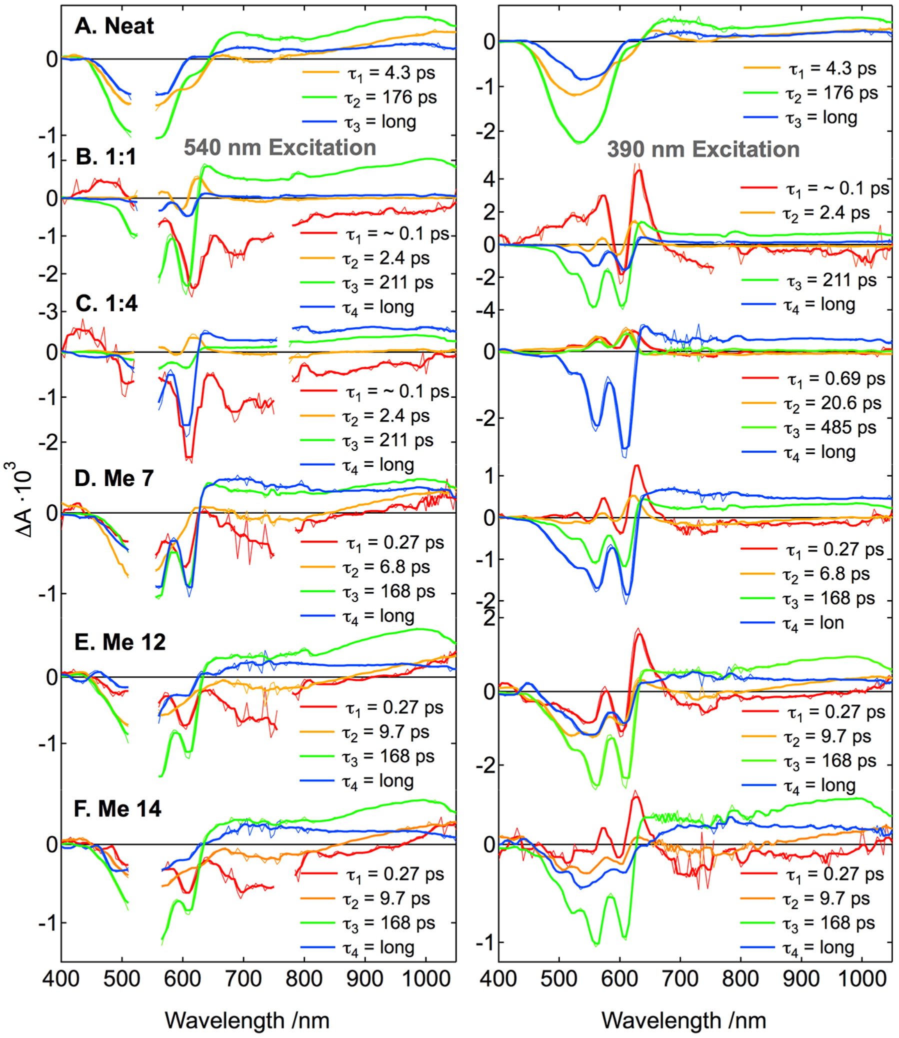

Supplementary Figure 2. Amplitude spectra obtained by global analysis of the transient absorption (TA) data. The amplitude spectra associated with the time constants shown in the legends, obtained by global analysis of the TA data with the sum of exponential functions, are shown for A) neat pBTTT, B) pBTTT:PCBM (1:1 weight ratio), C) pBTTT:PCBM (1:4 weight ratio), D) pBTTT:PCBM (1:1, processed with Me 7), E) pBTTT:PCBM (1:1, processed with Me 12) and F) pBTTT:PCBM (1:1, processed with Me 14). The data on the left side was recorded with excitation at 540 nm, and the one on the right side with excitation at 390 nm. Thicker solid lines were smoothed and overlaid to the raw amplitude spectra.

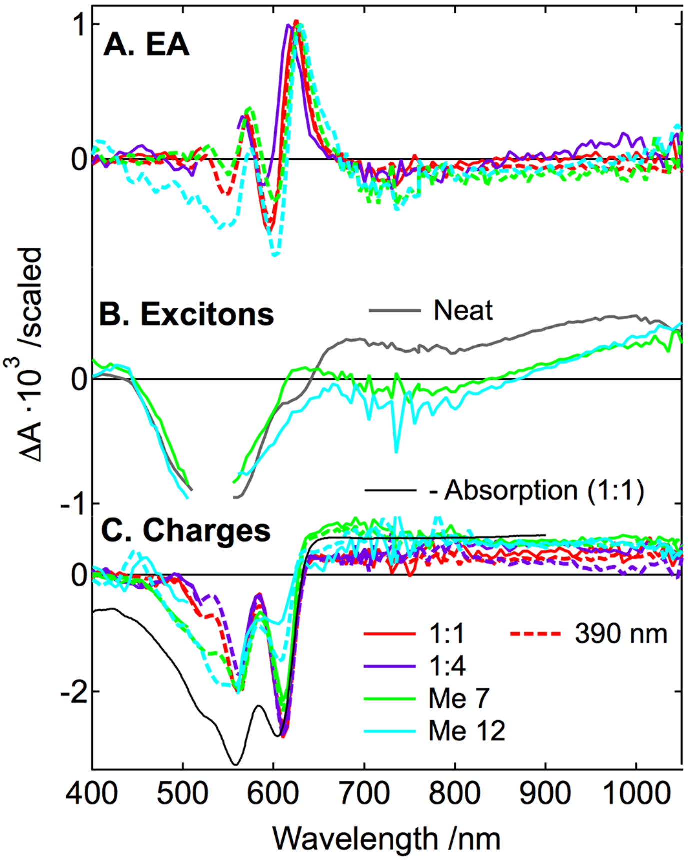

Supplementary Figure 3. Spectral components used for the decomposition of the transient absorption spectra. A) Electro-absorption (EA) signature, B) signature of pBTTT excitons and C) signature of charges in the pBTTT:PCBM blends. They were obtained for the different samples (pBTTT, pBTTT:PCBM and pBTTT:additive:PCBM) based on the amplitude spectra from the global analysis, as explained in the Supplementary Note 1. Solid lines are for excitation at 540 nm, while dashed lines are for excitation at 390 nm. The steady-state absorption spectrum of the 1:1 blend is also shown; its onset matches the onset of the ground state bleaching in the components corresponding to the charges (after subtraction of the EA).

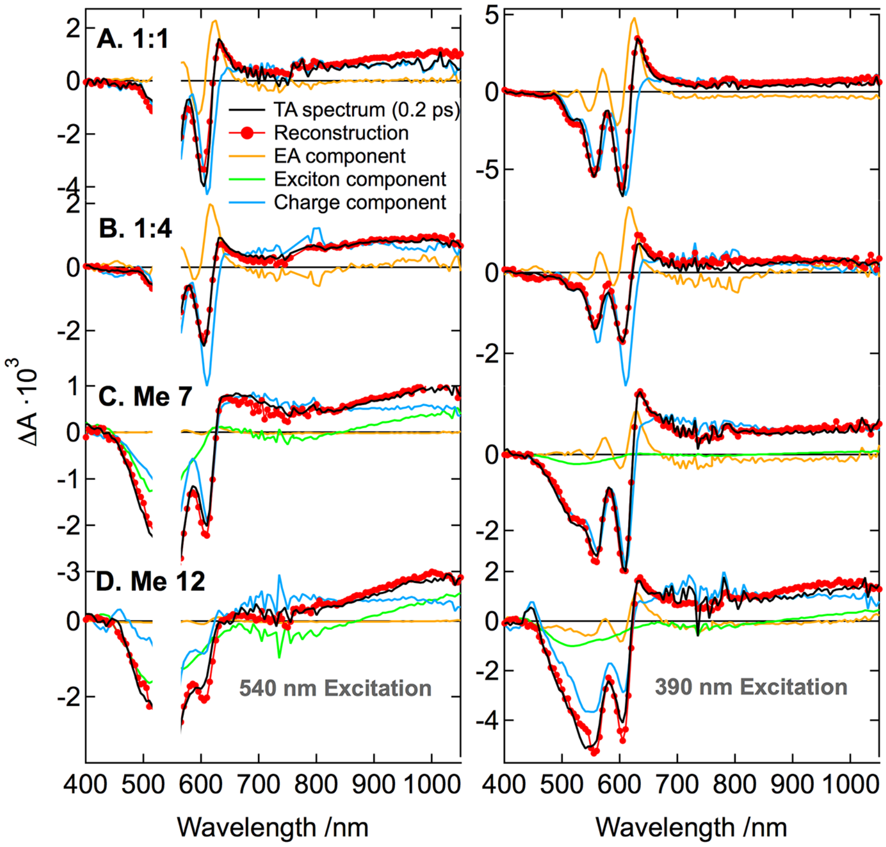

Supplementary Figure 4. Result of the spectral decomposition of the transient absorption (TA) spectra at 0.2 ps. A) pBTTT:PCBM (1:1 weight ratio), B) pBTTT:PCBM (1:4 weight ratio), C) pBTTT:PCBM (1:1, processed with Me 7) and D) pBTTT:PCBM (1:1, processed with Me 12). The experimental TA spectra (black lines) recorded at 0.2 ps following photo-excitation at 540 nm (left side) or 390 nm (right side) were decomposed into their spectral components, scaled by their corresponding coefficients: Electro-absorption (EA, orange line), pBTTT excitons (green line) and charges (blue line). The red line with markers is the reconstructed spectrum (sum of the scaled components). The results for the Me 14 sample are not shown, since they were similar to the ones with Me 12.

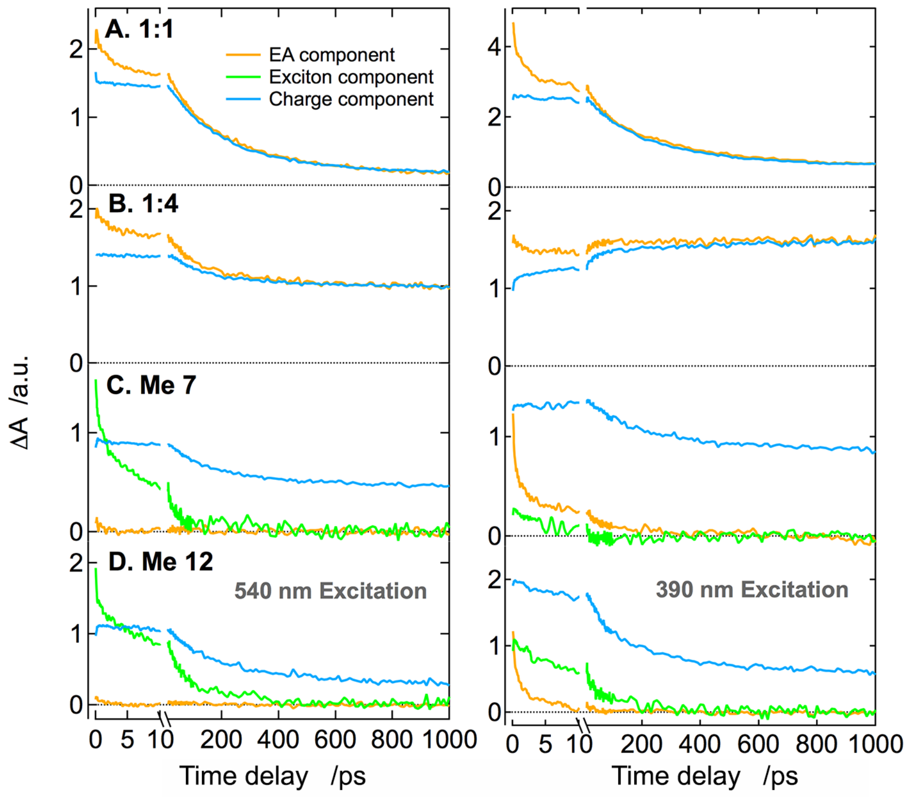

Supplementary Figure 5. Time profiles of the coefficients corresponding to the spectral components used to decompose the transient absorption spectra. The evolution of the electro-absorption (EA) signature (orange), the exciton signature (green) and the charge signature (blue) is shown, obtained from the spectral decomposition for A) pBTTT:PCBM (1:1 weight ratio), B) pBTTT:PCBM (1:4 weight ratio), C) pBTTT:PCBM (1:1, processed with Me 7) and D) pBTTT:PCBM (1:1, processed with Me 12). The data on the left side was recorded with excitation at 540 nm, and the one on the right side with excitation at 390 nm.

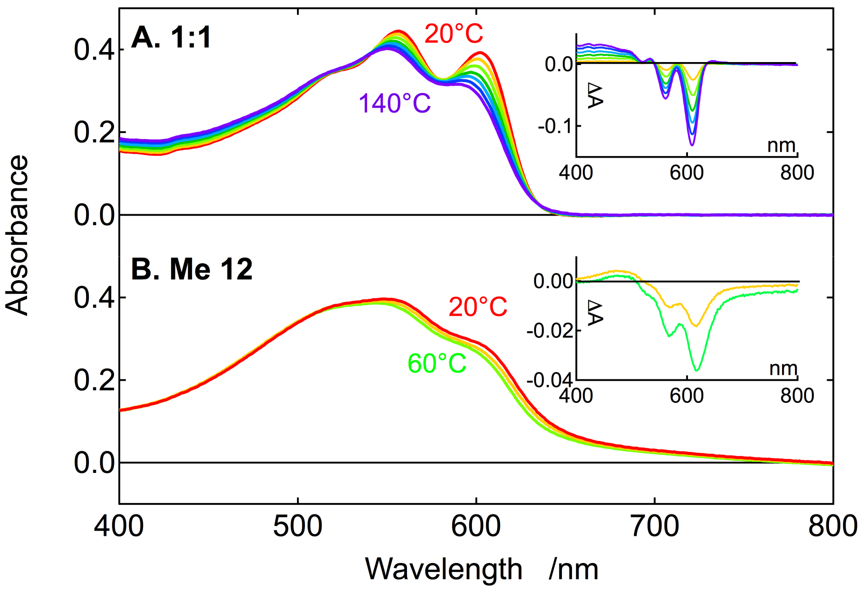

Supplementary Figure 6. Temperature dependence of the absorption spectra. Steady-state absorption spectra of A) pBTTT:PCBM (1:1 weight ratio, fully intercalated), and B) pBTTT:PCBM (1:1, processed with Me 12, predominantly phase-separated), were recorded at different temperatures using a Peltier temperature-controlled sample holder (FLASH-300, Quantum Northwest). Temperature steps of 20°C are shown and the spectral changes were reversible upon cooling. For the Me 12 sample, heating was stopped at 60°C due to sample degradation. The insets represent the differential temperature response obtained by subtracting the spectrum at 20°C from the ones at the other temperatures (starting at 40°C, orange line).

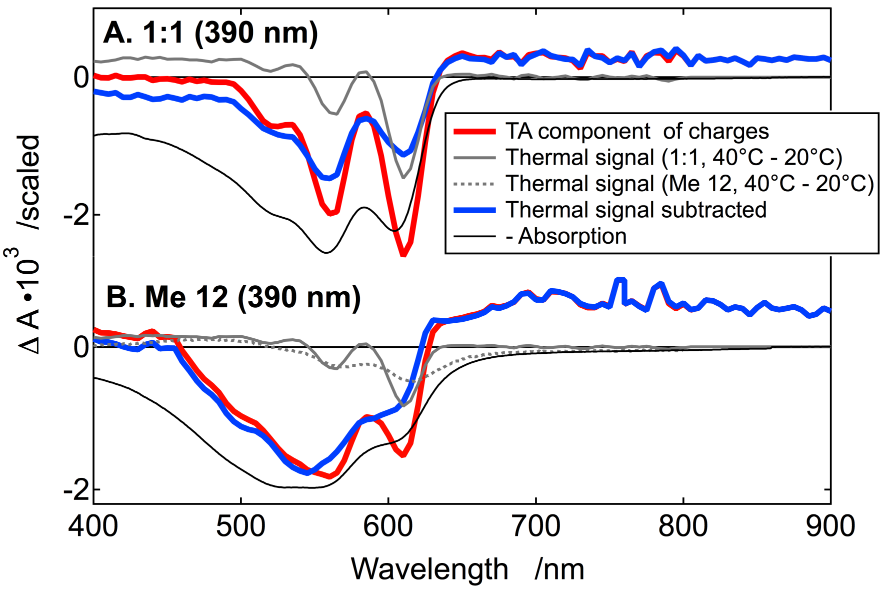

Supplementary Figure 7. Correction of the thermal response in the transient absorption (TA) data. The spectral component representing the signature of charges (same as in Supplementary Figure 3C), extracted from the TA data of A) pBTTT:PCBM (1:1 weight ratio, fully intercalated), and B) pBTTT:PCBM (1:1, processed with Me 12, predominantly phase-separated), both with 390 nm excitation, is shown in red without any correction, while the scaled thermal response (shown in grey and obtained from the 40°C - 20°C steady state absorption of the intercalated 1:1 blend) was subtracted to obtain the corrected blue curves. The shape of the ground state bleaching (GSB) becomes closer to the absorption (black curve).

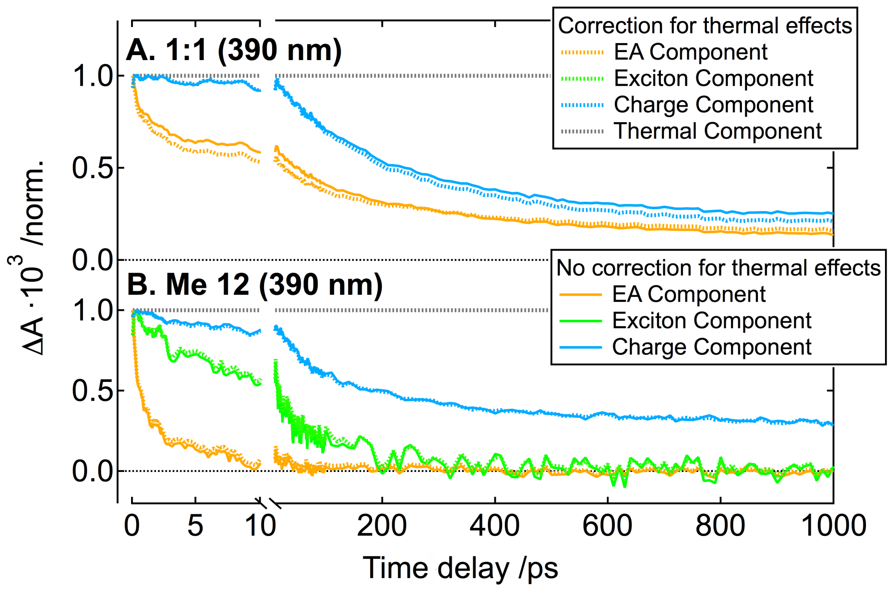

Supplementary Figure 8. Effect of the thermal response on the temporal evolution of the spectral components in the transient absorption (TA) data. Time profiles of the coefficients corresponding to the spectral components of the electro-absorption signature (EA, orange), the exciton signature (green) and the charge signature (blue), obtained from the spectral decomposition of the TA data for A) pBTTT:PCBM (1:1 weight ratio, fully intercalated), and B) pBTTT:PCBM (1:1, processed with Me 12, predominantly phase-separated), both with 390 nm excitation, are shown. The dotted lines represent the analysis taking into account the thermal artefact (by including the thermal modulation as an additional constant-amplitude component, and using the corrected shape of the charge component). The difference compared to the analysis without correction (solid lines) is negligible.

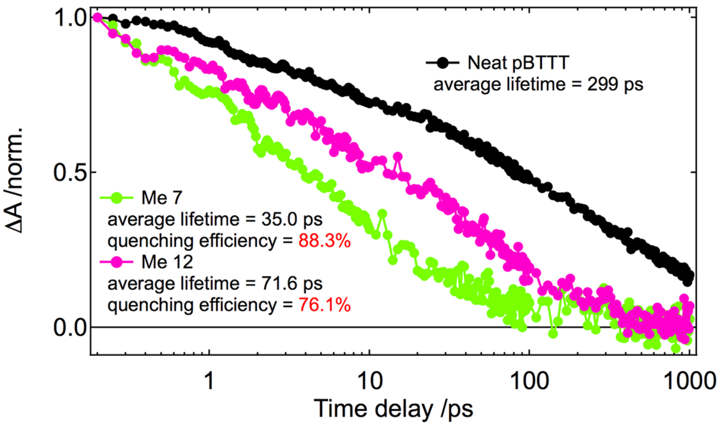

Supplementary Figure 9. Estimation of the quenching efficiency in the phase-separated samples (from transient absorption (TA) spectroscopy). Normalized time profiles of the exciton component obtained for neat pBTTT and the pBTTT:PCBM (1:1 by weight) blends processed with Me 7 or Me 12, from the decomposition of the TA spectra (540 nm excitation), are depicted. The average lifetime was estimated by integrating the dynamics und used to calculate the delayed quenching efficiency.

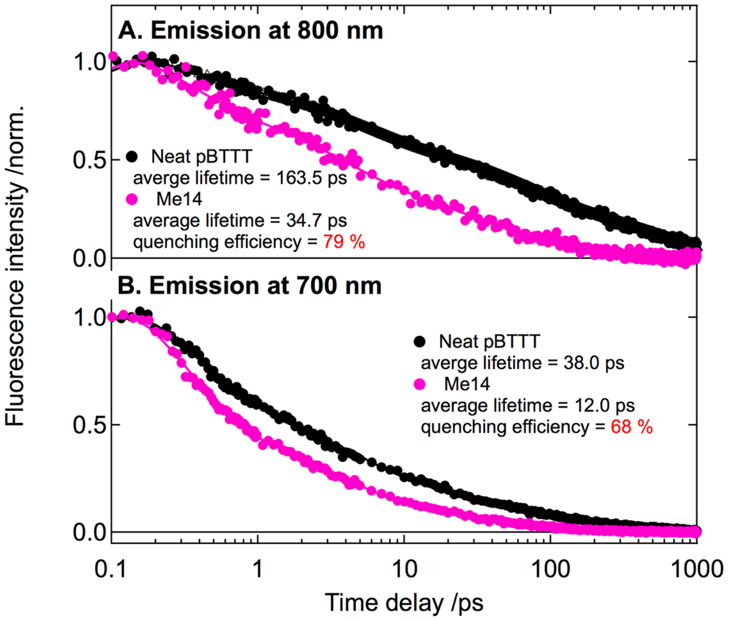

Supplementary Figure 10. Estimation of the quenching efficiency in the phase-separated samples (from fluorescence up-conversion spectroscopy). Normalized fluorescence time profiles obtained for neat pBTTT and the pBTTT:PCBM (1:1 by weight) blend processed with Me 14, at emission wavelengths of 700 nm and 800 nm, following excitation at 500 nm, are shown. The average lifetime was estimated from the integral of the dynamics und used to calculate the delayed quenching efficiency.

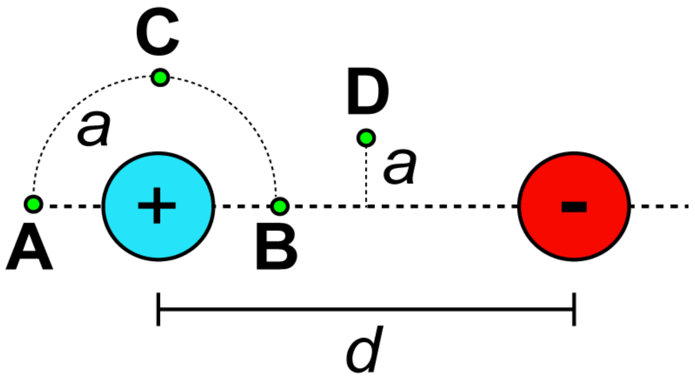

Supplementary Figure 11. Schematic representation of the different positions (A, B, C and D) with respect to an electron-hole dipole separated by distance d. Vector addition allowed to calculate the electric field magnitude at the four positions (see Supplementary Note 4).[1]

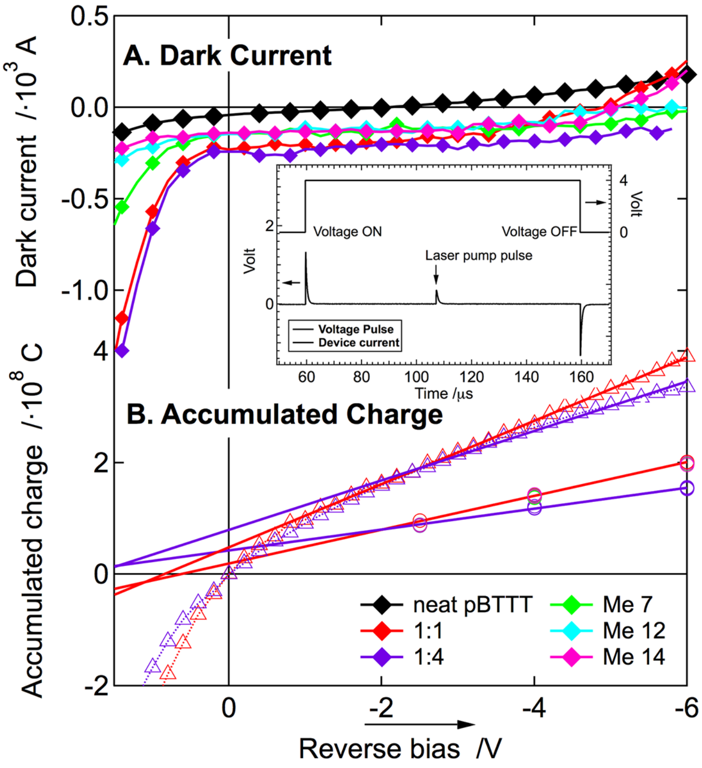

Supplementary Figure 12. Dark current and charge accumulation upon pulsed bias application to the investigated devices. A) Dark current as a function of applied reverse bias measured in the absence of illumination for the different pBTTT and pBTTT:PCBM devices. B) Charge accumulated upon switching on the reverse voltage pulse (obtained by integrating the corresponding current transient after subtracting the dark current) as a function of applied reverse bias. The triangles represent pBTTT:PCBM 1:1 and 1:4 devices with a 0.2 cm^2 electrode area, while the circles show devices with a 0.1 cm^2 electrode area (for all samples, but the additive data completely overlaps with the data for the 1:1 blend). Straight lines are a linear fit in the -2 V to -6 V range. The inset shows the typical current response, measured with an oscilloscope via a 50 Ω series load.

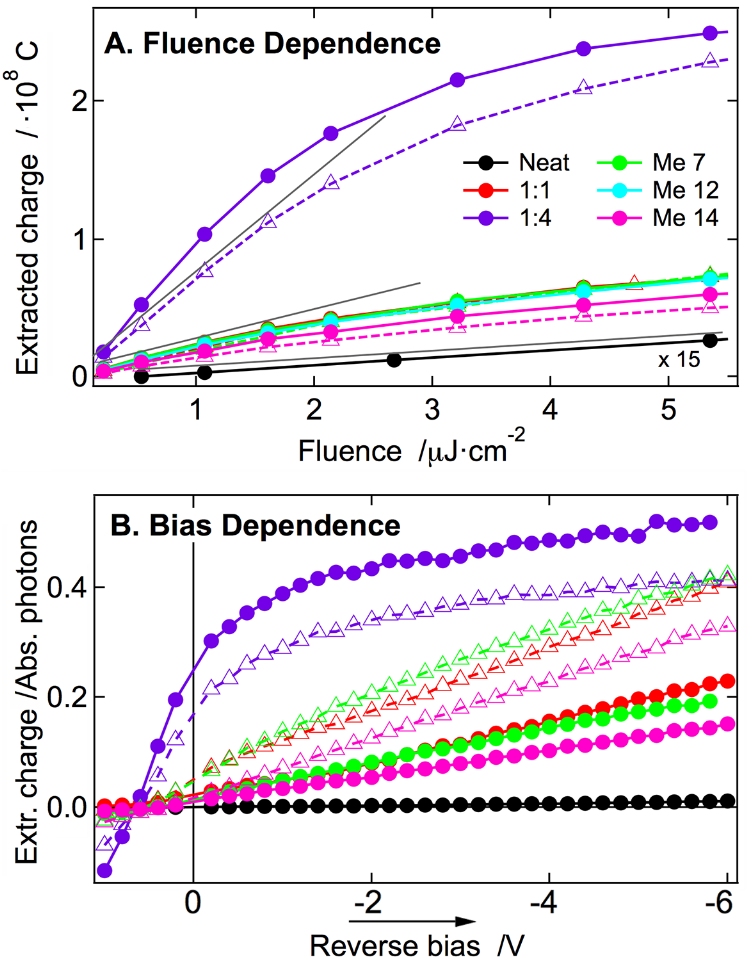

Supplementary Figure 13. Extracted charge (from the photocurrent) as a function of excitation fluence and applied bias. A) Extracted charge as a function of pump fluence (circles for excitation at 540 nm, triangles for excitation at 390 nm) for the different pBTTT and pBTTT:PCBM devices under -2.5 V reverse bias. Grey lines are added as guides to the eye. B) Extracted charge, scaled by the number of absorbed photons, as a function of applied reverse bias. Extracted charge was obtained by integrating the photocurrent transients measured with an oscilloscope via a 50 Ω series load. Data is shown for devices with a 0.1 cm^2 electrode area and a pump fluence within the linear photocurrent regime was used.

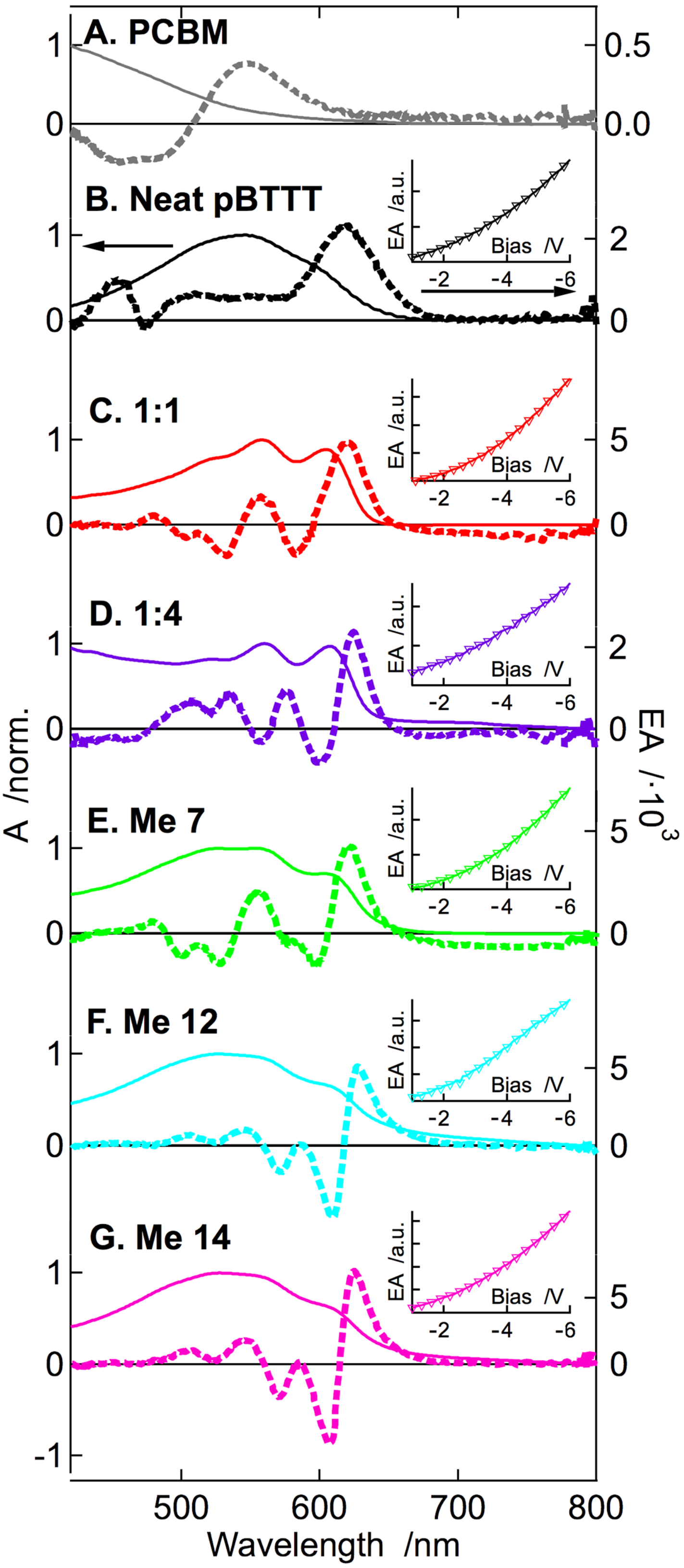

Supplementary Figure 14. Steady-state electro-absorption spectra. Steady-state absorption spectra (solid lines) and electro-absorption (EA) spectra (at -6 V, dashed lines), recorded for the neat pBTTT and PCBM samples and their blends are shown (insets: bias dependence of the EA amplitude around 620 nm).

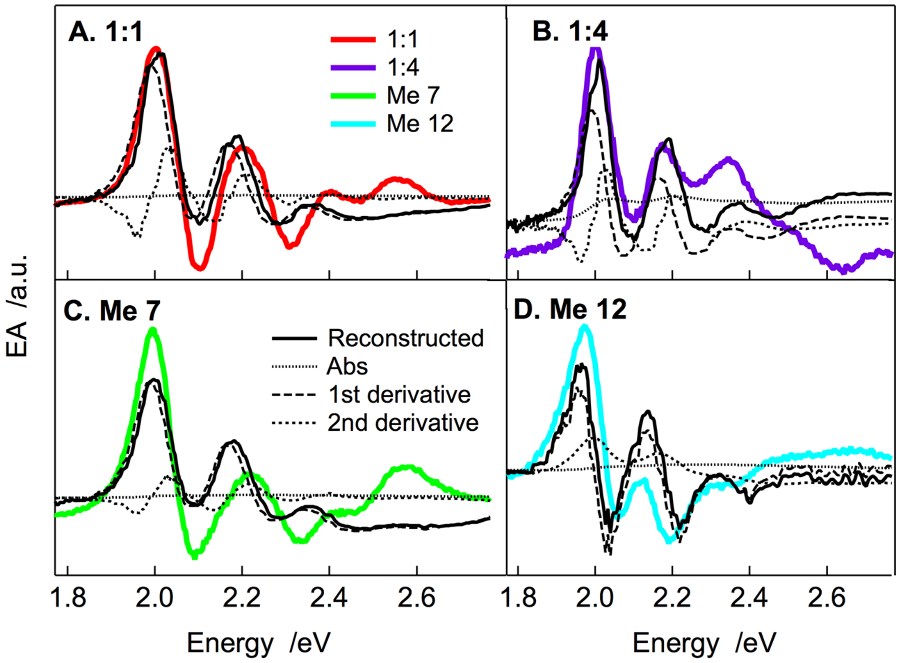

Supplementary Figure 15. Spectral decomposition of the experimental electro-absorption (EA) spectra recorded at -6 V. A) pBTTT:PCBM (1:1 weight ratio), B) pBTTT:PCBM (1:4 weight ratio), C) pBTTT:PCBM (1:1, processed with Me 7) and D) pBTTT:PCBM (1:1, processed with Me 12). The experimental data is shown in colour for the different samples, together with the scaled components (steady-state absorption spectrum, its first derivative and its second derivative) as well as their sum (reconstructed spectrum).

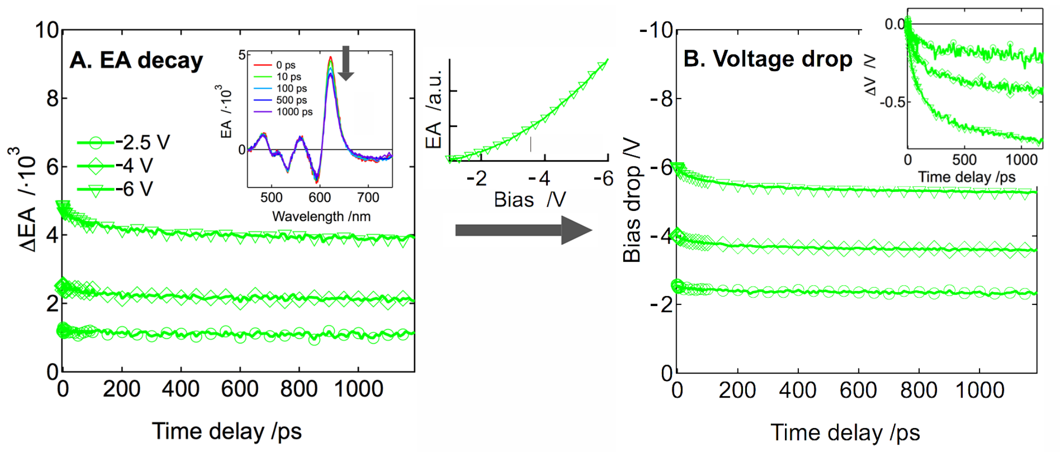

Supplementary Figure 16. Conversion of the electromodulated differential absorption (EDA) dynamics to a voltage drop across the devices. A) EDA dynamics measured for a device containing pBTTT:PCBM (1:1 by weight, processed with Me 7, 0.2 cm^2 electrode area), excited at 540 nm with a fluence of 1.0 $\mu J/cm^2$, under applied reverse biases of -2.5 V, -4 V or -6 V. The inset shows the decay of the electro-absorption (EA) spectrum at different time delays following pump excitation (-6 V bias), while the dynamics of the EA decay in the 620 nm peak are shown in the main figure. B) Using the relation between the steady-state EA amplitude around 620 nm and the applied reverse bias (middle inset), the EDA dynamics were translated to the bias drop across the solar cell, caused by the transport of charge carriers to the electrodes. Finally, the externally applied bias was subtracted, in order to get the voltage drop (ΔV, right inset).

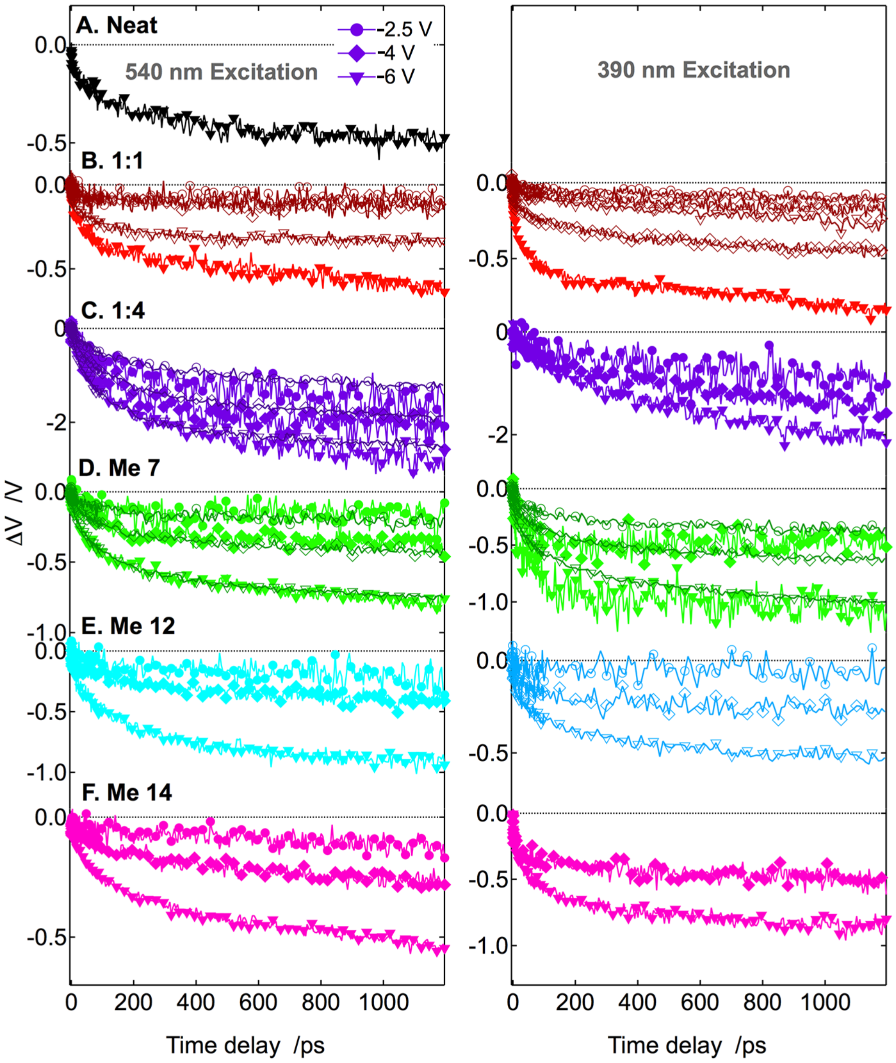

Supplementary Figure 17. Voltage drop dynamics obtained by electromodulated differential absorption (EDA) spectroscopy. The voltage drop (ΔV) for all the investigated devices, at reverse biases of -2.5 V (circles), -4 V (diamonds) or -6 V (triangles), upon excitation at 540 nm (left side) or 390 nm (right side), is shown. The lighter curves with full symbols were recorded on 0.1 cm^2 devices, while the darker curves with empty symbols were recorded on 0.2 cm^2 devices. A) Neat pBTTT (-6 V, 5.3 μJ/cm^2 excitation at 540 nm, 0.1 cm^2 device); B) pBTTT:PCBM (1:1 weight ratio, 1.6 μJ/cm^2 excitation at 540 nm for the 0.1 cm^2 device, 1.0 μJ/cm^2 excitation at 540 nm for the 0.2 cm^2 device, 4.4 μJ/cm^2 excitation at 390 nm for the 0.1 cm^2 device, 2.4 μJ/cm^2 and also 4.4 μJ/cm^2 excitation (only at -4 V) at 390 nm for the 0.2 cm^2 device); C) pBTTT:PCBM (1:4 weight ratio, 1.0 μJ/cm^2 excitation at 540 nm for both the 0.1 cm^2 and 0.2 cm^2 devices, 1.2 μJ/cm^2 excitation at 390 nm for the 0.1 cm^2 device); D) pBTTT:PCBM (1:1 processed with Me 7, 1.6 μJ/cm^2 excitation at 540 nm for the 0.1 cm^2 device, 1.0 μJ/cm^2 excitation at 540 nm for the 0.2 cm^2 device, 3.5 μJ/cm^2 excitation at 390 nm for the 0.1 cm^2 device, 2.4 μJ/cm^2 excitation at 390 nm for the 0.2 cm^2 device); E) pBTTT:PCBM (1:1 processed with Me 12, 1.6 μJ/cm^2 excitation at 540 nm for the 0.1 cm^2 device, 2.4 μJ/cm^2 excitation at 390 nm for the 0.2 cm^2 device); and F) pBTTT:PCBM (1:1 processed with Me 14, 1.6 μJ/cm^2 excitation at 540 nm for the 0.1 cm^2 device, 3.5 μJ/cm^2 excitation at 390 nm for the 0.1 cm^2 device).

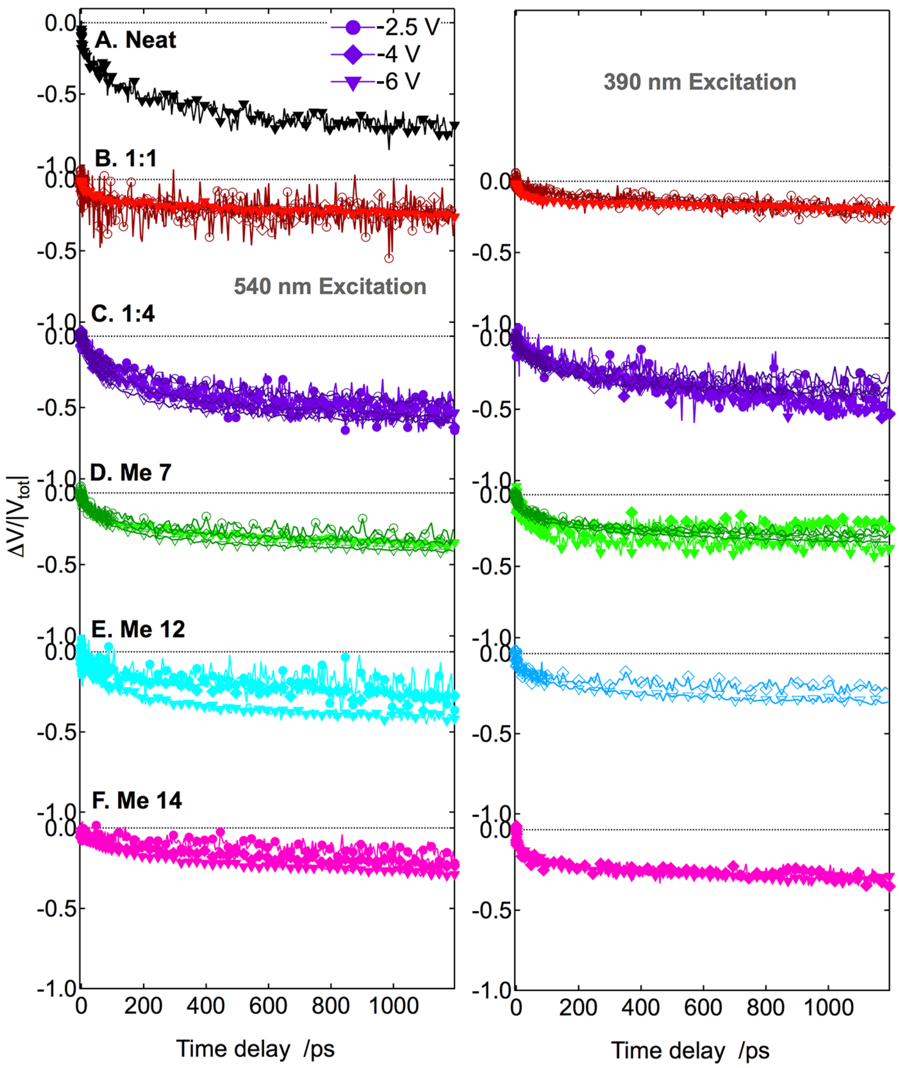

Supplementary Figure 18. Normalized voltage drop dynamics obtained by electromodulated differential absorption (EDA) spectroscopy. Voltage drop for the same devices and measurements as shown in Supplementary Figure 17, normalized by the total voltage drop calculated from the device capacitance and extracted photo-generated charge.

Supplementary Note 1. Spectral decomposition of the transient absorption data

The transient absorption (TA) spectra of the neat pBTTT, pBTTT:PCBM and pBTTT:additive:PCBM samples, excited at either 390 nm or 540 nm, are shown in **Supplementary Figure 1**.[1] They were globally analysed by fitting the sum of exponential functions to the dynamics at all probe wavelengths. This allowed estimating the time scales on which the excited-state processes occurred. The amplitude spectra (pre-exponential factors as a function of wavelength) are shown in **Supplementary Figure 2**. As explained in detail elsewhere,[1] mainly signatures due to charges and due to electro-absorption (EA) were present in the 1:1 and 1:4 blends processed without additives. The latter was caused by a Stark shift in the absorption spectra of polymer segments subject to local electric fields due to the vicinity of photo-generated charges. In the samples processed with the Me 7, Me 12 and Me 14 additives, the signature of slowly quenched excitons, similar to the one observed in neat pBTTT, was present in addition to the charges and EA (mainly at 540 nm excitation).

In order to separately follow the time evolution of the different contributions present in the TA spectra (excitons, charges and EA), the TA spectra at all time delays were decomposed into a linear combination of their components using a linear least squares fitting procedure ($\mathbf{A}$ is the TA spectrum at a given time delay, $\mathbf{B}$ is a matrix containing the spectral components, and $\mathbf{C}$ is a vector of the linear coefficients of the components):

$$\mathbf{A} = \mathbf{C} \cdot \mathbf{B} \qquad \textit{Supplementary Equation 1}$$

$$\mathbf{C} = (\mathbf{B}^{\mathrm{T}} \cdot \mathbf{B})^{-1} \cdot \mathbf{B}^{\mathbf{T}} \cdot \mathbf{A} \qquad \textit{Supplementary Equation 2}$$

Since knowing the spectral components was a pre-requisite of this analysis, they were estimated on the basis of the amplitude spectra obtained from the global analysis (**Supplementary Figure 2**). For neat pBTTT, the spectrum associated with τ_2 was used to represent the signature of the excitons, neglecting the weak contributions of spectral shifts and polaron or triplet states. For the additive samples excited at 540 nm, the τ_2-associated spectrum strongly resembled the signatures observed in neat pBTTT, so that is was used as the spectral component of excitons in those blends. Any contribution of PCBM excitons was neglected (their transitions are much weaker than the ones of the polymer and their absorption is flat).[2] In all the pBTTT:PCBM blends at both excitation wavelengths, the long-lived spectrum associated with τ_4 was used to obtain the component of the charges. It was dominated by the signature of positive pBTTT polarons. We did not distinguish between bound and free charges, since their TA signals are typically quite similar. In contrast to the additive samples, the EA signature was present in the long-lived τ_4 amplitude spectrum of the 1:1 and 1:4 blends and needed to be subtracted. Before the subtraction, the EA spectrum was scaled in a way so that the onset of the ground state bleaching (GSB) in the resulting charge spectrum (without EA) coincided with the absorption onset of the steady-state spectrum. The EA spectral component, resembling the oscillatory first derivative of the absorption, could be clearly identified as the τ_2 amplitude spectrum of the 1:1 blend (390 nm and 540 nm excitation) and of the 1:4 blend (540 nm excitation), as well as the τ_1 amplitude spectrum of the additive samples excited at 390 nm.

The spectral components obtained in this way are summarised for the different samples in **Supplementary Figure 3**. Their absolute amplitude is not meaningful, since their absorption coefficient could not be precisely determined due to uncertainty in the excitation density

caused by sample inhomogeneity. Therefore, the spectral components were simply normalized for better comparison between the different samples. The decomposition of the TA spectra recorded 0.2 ps after excitation at 390 nm or 540 nm is shown in **Supplementary Figure 4** (experimental spectrum, components scaled by the corresponding linear coefficient, and their sum yielding the reconstructed spectrum). The analysis was repeated at all pump-probe time delays, and the time profile of the coefficients represents the evolution of the exciton, charge and EA populations (**Supplementary Figure 5**).

<u>Supplementary Note 2.</u> Thermal modulation artefacts in the transient absorption data

It can be seen in **Supplementary Figure 3C** that the negative part of the transient absorption (TA) components assigned to charges (below 630 nm) does not correspond exactly to the corresponding steady-state absorption spectra of the investigated blends, in contrast to what would be expected for a ground state bleaching (GSB) signature. This has been explained in terms of overlapping positive TA bands,[2] changes in the delocalization of the charges,[3,4] or migration of charges to regions in the blend with different absorption.[5,6] There is also the possibility of a thermal modulation artefact, caused by the transient heating of the thin films after thermalization of the photo-excited states.[7,8] In order to investigate whether this takes place here, we have measured the temperature-dependence of the steady-state absorption spectra for the fully intercalated 1:1 blend and the phase-separated sample processed with Me 12 (**Supplementary Figure 6**). Indeed, we find a thermochromic effect (reversible upon cooling) and have calculated the differential temperature response by subtracting the spectrum at 20°C from the ones at the other temperatures (insets of **Supplementary Figure 6**). This differential temperature response scales linearly in amplitude with temperature and its shape is essentially temperature-independent. It corresponds approximately to the second derivative of the absorption at 20°C in the case of the 1:1 blend (and is thus clearly distinct from the EA signature seen in the TA data), while it corresponds roughly to the first derivative of the absorption at 20°C for the sample treated with Me 12. In the latter, the situation is complicated by the presence of several phases with potentially different temperature dependence.

Since thermal modulation artefacts in the TA data, caused by heating due to the pump beam, are expected to have a signature resembling the differential temperature response measured in steady-state absorption,[7] we subtracted the latter (after appropriate scaling) from the TA spectra corresponding to the charge component for the 1:1 and Me 12 samples excited at 390 nm. Indeed, the shape of the GSB becomes much closer to the steady-state absorption spectrum when the structured thermal signal is removed, while the positive absorption band of the charges (above 630 nm) is not affected (**Supplementary Figure 7**). This shows that thermal modulation artefacts are indeed present and distort the negative GSB signature of the TA spectra in the pBTTT:PCBM blends. From the amplitude of the thermal modulation ($\sim 10^{-3}$), we estimate a temperature increase of 0.5-1°C in the photo-excited samples. We note that in the case of the sample treated with Me 12, we subtracted the differential thermal response measured in the 1:1 sample (not in the Me 12 sample) in order to obtain a reasonable shape of the GSB. This is consistent with selective excitation into co-crystal regions at 390 nm and predominant heat dissipation into those regions of the film. In order to evaluate how much the thermal modulation affects our analysis of the TA data by spectral decomposition (Supplementary Note 1), we repeated the analysis for the 1:1 and Me 12 samples with 390 nm excitation, including the thermal modulation as an additional component, and using the corrected shape of the charge component (without thermal modulation). Since heat dissipation in polymer thin films has been shown to occur on the tens of nanosecond time scale or slower,[7,8] we fixed the contribution of the thermal modulation to a constant amplitude within the investigated 1 ns time scale. We find that the temporal evolution of the charge, exciton and EA signatures in the fully intercalated 1:1 blend and phase-separated Me 12 sample are hardly affected by taking into account the thermal modulation (**Supplementary Figure 8**). In view of the negligible effect, we have decided not to systematically correct all TA data for thermal modulation artefacts, also to avoid an unnecessary amount of data manipulation.

<u>Supplementary Note 3.</u> Delayed quenching efficiency in the phase-separated samples

The pBTTT:PCBM blends processed with the additives contained neat pBTTT domains. Upon photo-excitation of the polymer in the transient absorption (TA) experiments, some excitons in those samples underwent prompt dissociation into charges (in the intermixed co-crystal regions and at the edge of neat domains), but others first had to diffuse out of the neat pBTTT regions to a PCBM quenching site, leading to delayed exciton quenching. The following analysis was carried out in order to determine the fraction of excitons lost in the neat domains due to their natural decay to the ground state before reaching a PCBM. The average lifetime of the excitons without quencher (τ_{av}, from the neat pBTTT sample) and in the presence of quencher ($\tau_{av,Q}$, from the pBTTT:PCBM blends processed with Me 7 or Me 12, 540 nm excitation) was estimated by integrating the normalized time profiles of the exciton component obtained from the decomposition of the TA data (**Supplementary Figure 9**). Note that due to the normalization, the prompt quenching (faster than the experimental time resolution) was not taken into account. Values of 299 ps, 35 ps and 72 ps were found, respectively, implying a delayed quenching efficiency, η_{del}^{Q}, of 88% for the blend processed with Me 7, and of 76% for the one with Me 12:

$$\eta_{del}^{Q} = 1 - \frac{\tau_{av,Q}}{\tau_{av}} = 1 - \frac{\int \Delta A_{ex}^{Q} dt}{\int \Delta A_{ex} dt} \qquad \textit{Supplementary Equation 3}$$

High-energy and low-energy excitons in the inhomogeneous exciton population of pBTTT could be targeted separately using fluorescence-up conversion spectroscopy at different emission wavelengths (**Supplementary Figure 10**). Following a similar approach as for the exciton dynamics from TA spectroscopy, a delayed quenching efficiency of 79 % was found for low energy excitons emitting at 800 nm for pBTTT:PCBM processed with Me 14 (equivalent to Me 12), which dropped to 68 % for high-energy excitons emitting at 700 nm.

Supplementary Note 4. **Electrostatic simulations of the local electric field strength**

In order to estimate the local electric field felt by polymer segments in the vicinity of the photo-generated charges as a function of electron-hole separation, a simple point-charge electrostatic model (which we previously described in detail) was used.[1] The electro-absorption (EA) signature most visible in the transient absorption (TA) spectra results from transitions in pBTTT, so that we limited the analysis to the effect of photo-generated pBTTT holes on neighbouring polymer segments located 0.2 nm away from the hole (an estimate based on π-stacking distance and typical size of polymer segments). Different geometric locations with respect to the electron-hole dipole were considered (A - aligned with the dipole on the other side of the hole; B - aligned with the dipole between the charges; C - perpendicular to the dipole next to the hole; D - in the central plane dividing the dipole). They are depicted in **Supplementary Figure 11**, and the magnitude of the electric field, $|\vec{E}|$, at the four positions was calculated using vector addition, where a = 0.2 nm is the distance from the hole, $e = 1.6\cdot10^{-19}$ C is the unit charge, $\varepsilon_0 = 8.85\cdot10^{-12}$ As/Vm is the permittivity of free space, and d is the electron-hole separation:

$$\left|\vec{E}^A\right| = \frac{e}{4\pi\varepsilon_0}\left(\frac{1}{a^2} - \frac{1}{\left(d+a\right)^2}\right)$$ *Supplementary Equation 4*

$$\left|\vec{E}^B\right| = \frac{e}{4\pi\varepsilon_0}\left(\frac{1}{a^2} + \frac{1}{\left(d-a\right)^2}\right)$$ *Supplementary Equation 5*

$$\left|\vec{E}^C\right| = \frac{e}{4\pi\varepsilon_0}\sqrt{\left(\frac{1}{a^2} - \frac{a}{\left(a^2+d^2\right)^{3/2}}\right)^2 + \left(\frac{d}{\left(a^2+d^2\right)^{3/2}}\right)^2}$$ *Supplementary Equation 6*

$$\left|\vec{E}^D\right| = \frac{e}{4\pi\varepsilon_0}\frac{d}{\left(\frac{d^2}{4}+a^2\right)^{3/2}}$$ *Supplementary Equation 7*

$$\left|\vec{E}_{free}\right| = \frac{e}{4\pi\varepsilon_0}\left(\frac{1}{a^2}\right)$$ *Supplementary Equation 8*

Supplementary Note 5. Current response of the investigated devices

During electromodulated differential absorption (EDA) measurements on full solar cell devices, the current response was recorded via a 50 Ω series load with an oscilloscope. When the devices were reverse-biased (down to -6 V) by applying the external voltage pulse, charge injection was minimal, as seen in **Supplementary Figure 12A** (dark current as a function of applied bias). In this bias regime, the devices acted as dielectric capacitors, meaning that a homogeneous electric field of the order of 10^5 V/cm was created across their active polymer:fullerene layer. When the square voltage pulse was switched on/off, a corresponding positive/negative current response related to the displacement of accumulating/de-accumulating charges was observed (inset of **Supplementary Figure 12**). In strong reverse bias, the capacitance of organic solar cells is constant and given by the geometric value C_g,[9] where ε is the dielectric constant of the organic material, A is the electrode area (0.1 cm^2 or 0.2 cm^2), and d is the active layer thickness (~100 nm):

$$C_g = \frac{\varepsilon_0 \varepsilon A}{d}$$ *Supplementary Equation 9*

Indeed, the accumulated charge ($q_{acc} = \int I_{acc}(t)dt$, obtained by integrating the current transient observed when the voltage pulse was switched on), varied linearly with the applied bias in the -2 V to -6 V range (**Supplementary Figure 12B**). From the slope, a capacitance of 3.0 nF for all the 1:1 pBTTT:PCBM cells with or without additives was found for the 0.1 cm^2 devices (5.7 nF for the 0.2 cm^2 devices). For the 1:4 blend, a capacitance of 1.9 nF was obtained (4.4 nF for the larger device). These values correspond very well to the ones expected for organic thin films with a dielectric constant of ~2-3.

When the pump pulses excited the devices while the external reverse bias was on, an additional photocurrent transient was seen on the oscilloscope ($I_{photo}(t)$). The extracted photo-generated charge (q_{ext}) was obtained from the integral of the photocurrent transient, after subtraction of the dark current. In **Supplementary Figure 13**, the extracted charge as a function of pump fluence (at a fixed reverse bias of -2.5 V) and as a function of applied bias (in the linear excitation regime), are shown. To account for variations in sample absorbance between the different blends and at the two excitation wavelengths, the data in **Supplementary Figure 13B** was scaled by the number of absorbed photons (Ph_{abs}), where e is the unit charge, h is Planck's constant, c is the speed of light, A is the electrode area, and T^{λ} is the transmittance of the sample at the excitation wavelength λ_{ex} (as measured between the electrodes of the device). Since the back-reflection on the electrode and any cavity interference effects were not considered, this does not represent the internal quantum efficiency, but merely serves a better sample comparison.

$$q_{ext} = \int I_{photo}(t)dt$$ *Supplementary Equation 10*

$$q_{ext}/Ph_{abs} = \frac{\int I_{photo}dt}{e \cdot (\lambda_{ex}/hc) \cdot Fluence \cdot (1 - T^{\lambda}) \cdot A}$$

Supplementary Equation 11

<u>Supplementary Note 6.</u> Analysis of the steady-state electro-absorption (EA) spectra

The steady-state EA spectra (recorded in the absence of pump excitation at an applied bias of -6 V), together with the steady-state absorption spectra of the corresponding devices (measured between the electrodes), are shown in **Supplementary Figure 14** for neat pBTTT, neat PCBM and the various pBTTT:PCBM blends with and without additives. We note that when probing in reflection geometry, the optical electric field of the probe ($|E_{opt}|^2$) is reduced towards the metal electrode. The observed EA signal was therefore due to a population of chromophores, which was higher towards the ITO side and lower at the metal side of the device. Assuming that the distribution of phase morphologies was homogeneous throughout the film thickness, we can nevertheless consider that the measured EA spectra represent the "bulk" response of the film.

The EA spectra could be quite well reproduced using a linear combination of the first and second derivatives of the corresponding steady-state absorption spectra, as well a minor contribution of the absorption spectrum itself (**Supplementary Figure 15**).[10] A similar procedure was used as for the decomposition of the TA spectra (Supplementary Note 1). Deviation from the experimental data can be explained by the inhomogeneous phase morphology of the blends, since the possibly different EA contributions from pBTTT in co-crystal regions, neat pBTTT, and PCBM were not separately addressed during the decomposition of the EA spectra. Also, the effect of the electric field on vibrational energies was not considered,[11] since the analysis of the EA lineshape was beyond the scope of the current investigation. In the insets of **Supplementary Figure 14**, the typically quadratic dependence of the EA amplitude around the ~620 nm maximum (obtained by integrating the peak in the ~615-635 nm region) on the applied reverse bias is depicted. A power function ($y = ax^b + c$, with b ≈ 0.5) or exceptionally a polynomial function ($y = ax^5 + bx^4 + cx^3 + dx^2 + ex + f$, e.g. Me 12 sample) was used to empirically characterize this dependence.

<u>Supplementary Note 7.</u> Analysis of the electromodulated differential absorption (EDA)

During time-resolved EDA measurements, the electro-absorption (EA) spectra were recorded after excitation of the sample with the pump. The transport of the photo-generated charges towards the electrodes shielded the externally applied reverse bias and thus reduced the electric field within the active layer of the device. This was directly measured with femtosecond resolution as a reduction in the EA amplitude at different time delays after excitation (**Supplementary Figure 16**). Knowing the empirical relation between the EA amplitude around 620 nm and the applied voltage (insets of **Supplementary Figure 14**), the reduction of the EA signal was directly translated to a time-resolved voltage drop ($\Delta V(t)$) across the solar cell. **Supplementary Figure 17** shows the voltage drop obtained for the investigated devices, for different applied biases and for the two excitation wavelengths (390 nm and 540 nm).

Applying a reverse bias can in principle induce a change in amplitude of the transient absorption (TA) features (ground state bleaching, charge signatures) due to modified pump absorption in the presence of the field, or lead to the appearance of new field-induced transient peaks. This would cause additional features in the differential EDA spectra that are different from bulk EA of the thin films. However, the EDA spectra that we measured (recorded with pump excitation) had the same shape as the EA spectra recorded without pump. In fact, the effect of the field on the absorbed pump intensity was negligible. At 540 nm, there was an increase of absorption of the order of 10^{-3} with an applied field of -6 V in all samples, corresponding to a minimal increase of absorbed pump photons of < 0.4%. Such a small increase in pump photons is not expected to cause any measurable transient response. Moreover, there was hardly any EA at 390 nm, so that the field affected the absorption at this excitation wavelength even less. We also want to point out that the "gradient" in EA response due to the node in the probe intensity when it is reflected off the metal electrode, did not affect the dynamics of the EDA. EDA is sensitive to the total electric field across the thin film, measured by means of the Stark effect and probed via the evolution of the bulk EA spectrum (and not by optically following individual charges). The diffusion of the charges in and out of the "blind zone" near the metal electrode does not cancel their effect on the total electric field, which is still reduced by their displacement and which still leads to a reduction of the bulk EA response.

When all the photo-generated charges were extracted under reverse bias, the capacitor formed by the solar cell (with accumulated charge $q_{acc} = \int I_{acc}(t)dt$, see Supplementary Note 5) was discharged by the amount of the extracted charge ($q_{ext} = \int I_{photo}(t)dt$):

$$q_{final} = q_{acc} - q_{ext} = \int I_{acc}(t)dt - \int I_{photo}(t)dt$$

Supplementary Equation 12

The total voltage drop ($|\Delta V_{tot}|$) expected upon full charge extraction could thus be estimated by dividing the integrated photocurrent by the device capacitance.[12] The value of $|\Delta V_{tot}|$, obtained in the conditions corresponding to each EDA measurement, was then used to normalize $\Delta V(t)$ ($\Delta V(t)/|\Delta V_{tot}|$, **Supplementary Figure 18**). For the normalization, we

used $C \approx \frac{q_{acc}}{V_{app}}$. Since the applied bias (V_{app}), neglecting the built-in field, was used for both the determination of $\Delta V(t)$ and $|\Delta V_{tot}|$, the overall effect of this approximation cancelled.

$$\frac{\Delta V(t)}{|\Delta V_{tot}|} = \frac{\Delta V(t) \cdot C}{\int I_{photo}(t)dt} \approx \frac{\Delta V(t) \cdot \int I_{acc}(t)dt}{\int I_{photo}(t)dt \cdot V_{app}}$$

Supplementary Equation 13

<u>Supplementary Note 8.</u> Calculation of the electron-hole separation in the devices

The average electron-hole separation of the photo-generated charges, $\langle l(t)\rangle$, in the direction perpendicular to the device plane, could be computed from the time-resolved voltage drop across the solar cells (where d is the active layer thickness):[13]

$$\langle l(t)\rangle = d\left(1-\sqrt{1-\frac{\Delta V(t)}{\left|\Delta V_{tot}\right|}}\right) \qquad \textit{Supplementary Equation 14}$$

The analysis assumes prompt charge generation, and was therefore not applicable to samples that showed delayed exciton dissociation. The above expression for $\langle l(t)\rangle$ will now be derived. The time evolution of the electric field change ($\Delta E(t)$) inside the device is related to the time-resolved number of extracted charges ($q_{ext}(t)$) and the device capacitance (C). It can alternatively be expressed in terms of the average separation distance $\langle l(t)\rangle$, the density of the free charge carriers ($n(t)$) and the dielectric constant of the active layer (ε):

$$\Delta E(t) = \frac{\Delta V(t)}{d} = \frac{q_{ext}(t)}{C\cdot d} = \frac{n(t)\cdot e\cdot\langle l(t)\rangle}{\varepsilon\cdot\varepsilon_0} \qquad \textit{Supplementary Equation 15}$$

If n_{tot} is the total concentration of free charge carriers, which are promptly photo-generated and all extracted in strong reverse bias (no bimolecular recombination), we obtain:

$$n(t) = n_{tot}\cdot\left(1-\frac{\langle l(t)\rangle}{d}\right) \qquad \textit{Supplementary Equation 16}$$

The above equations can then be combined to obtain the time-dependent photocurrent:

$$I_{photo}(t) = \frac{\partial q_{ext}(t)}{\partial t} = C\cdot d\frac{\partial\Delta E(t)}{\partial t} = A\cdot e\cdot n_{tot}\cdot\left(1-\frac{\langle l(t)\rangle}{d}\right)\frac{\partial l(t)}{\partial t}$$

Supplementary Equation 17

Here, the capacitance was replaced by its geometric value $C = \frac{\varepsilon_0\varepsilon A}{d}$. Integration of the time-dependent photocurrent now gives the number of charges transported by the photocurrent:

$$\Delta q(t) = \int I_{photo}(t)dt = A\cdot e\cdot n_{tot}\left(\langle l(t)\rangle - \frac{\langle l(t)\rangle^2}{2\cdot d}\right)$$

Supplementary Equation 18

Solving this quadratic equation for $\langle l(t)\rangle$ yields:

$$\langle l(t)\rangle = d\left(1\pm\sqrt{1-\frac{4}{2\cdot d\cdot A\cdot e\cdot n_{tot}}\cdot\Delta q(t)}\right) = d\left(1\pm\sqrt{1-\frac{\Delta q(t)}{\Delta q_{tot}}}\right)$$

Supplementary Equation 19

with $\Delta q_{tot} = \frac{1}{2} \cdot C \cdot d \cdot \Delta E_{tot} = \frac{1}{2} \cdot C \cdot d \cdot \frac{n_{tot} \cdot e \cdot \left[\langle l(t) \rangle = d\right]}{\varepsilon \cdot \varepsilon_0} = \frac{1}{2} \cdot A \cdot n_{tot} \cdot e \cdot d$

Supplementary Equation 20

The factor ½ arises from the fact that both electrons and holes contribute to the photocurrent. Finally, knowing that $\Delta q \propto C \cdot \Delta V$, and choosing the sign in front of the square root as negative (since the limits are $\langle l(t) \rangle$ = D when $\frac{\Delta V(t)}{|\Delta V_{tot}|}$ = 1, and $\langle l(t) \rangle$ = 0 when $\frac{\Delta V(t)}{|\Delta V_{tot}|}$ = 0):

$$\langle l(t) \rangle = d\left(1 - \sqrt{1 - \frac{\Delta V(t)}{\left|\Delta V_{tot}\right|}}\right)$$

Supplementary Equation 14

Supplementary References

1 Scarongella, M. *et al.* A close look at charge generation in polymer:fullerene blends with microstructure control. *J. Am. Chem. Soc.* **137**, 2908-2918, (2015).

2 Paraecattil, A. A. & Banerji, N. Charge separation pathways in a highly efficient polymer: fullerene solar cell material. *J. Am. Chem. Soc.* **136**, 1472-1482, (2014).

3 Hodgkiss, J. M. *et al.* Exciton-charge annihilation in organic semiconductor films. *Adv. Funct. Mater.* **22**, 1567-1577, (2012).

4 Howard, I. A., Mauer, R., Meister, M. & Laquai, F. Effect of morphology on ultrafast free carrier generation in polythiophene:fullerene organic solar cells. *J. Am. Chem. Soc.* **132**, 14866-14876, (2010).

5 Etzold, F. *et al.* Ultrafast exciton dissociation followed by nongeminate charge recombination in PCDTBT:PCBM photovoltaic blends. *J. Am. Chem. Soc.* **133**, 9469-9479, (2011).

6 Etzold, F. *et al.* The effect of solvent additives on morphology and excited-state dynamics in PCPDTBT:PCBM photovoltaic blends. *J. Am. Chem. Soc.* **134**, 10569-10583, (2012).

7 Rao, A., Wilson, M. W. B., Albert-Seifried, S., Di Pietro, R. & Friend, R. H. Photophysics of pentacene thin films: The role of exciton fission and heating effects. *Phys. Rev. B* **84**, (2011).

8 Albert-Seifried, S. & Friend, R. H. Measurement of thermal modulation of optical absorption in pump-probe spectroscopy of semiconducting polymers. *Appl. Phys. Lett.* **98**, 223304, (2011).

9 Fabregat-Santiago, F., Garcia-Belmonte, G., Mora-Sero, I. & Bisquert, J. Characterization of nanostructured hybrid and organic solar cells by impedance spectroscopy. *Phys. Chem. Chem. Phys.* **13**, 9083-9118, (2011).

10 Lanzani, G. *The photophysics behind photovoltaics and photonics*. (Wiley-VCH Verlag& Co, 2012).

11 Harrison, M. G. *et al.* Electro-optical studies of a soluble conjugated polymer with particularly low intrachain disorder. *Phys. Rev. B* **60**, 8650-8658, (1999).

12 Devizis, A. *et al.* Dissociation of charge transfer states and carrier separation in bilayer organic solar cells: A time-resolved electroabsorption spectroscopy study. *J. Am. Chem. Soc.* **137**, 8192-8198, (2015).

13 Pranculis, V. *et al.* Charge carrier generation and transport in different stoichiometry APFO3:PC61BM solar cells. *J. Am. Chem. Soc.* **136**, 11331-11338, (2014).
